# Synthesis and Biological Evaluation of 4β-N-Acetylamino Substituted Podophyllotoxin Derivatives as Novel Anticancer Agents

**DOI:** 10.3389/fchem.2019.00253

**Published:** 2019-04-24

**Authors:** Jinbao Wei, Jinghong Chen, Peijun Ju, Le Ma, Li Chen, Weidong Ma, Tao Zheng, Guangyi Yang, Yong-Xiang Wang

**Affiliations:** ^1^King's Lab, School of Pharmacy, Shanghai Jiao Tong University, Shanghai, China; ^2^Department of Pharmacy, Institute of Wudang Herbal Medicine Research, Taihe Hospital, Hubei University of Medicine, Shiyan, China; ^3^Shanghai Mental Health Center, School of Medicine, Shanghai Jiao Tong University, Shanghai, China; ^4^Baoan Hospital of Traditional Chinese Medicine, Shenzhen, China

**Keywords:** podophyllotoxin derivatives, C-4 substitutions, anticancer agent, biological evaluation, structure–activity relationships (SAR)

## Abstract

A series of novel podophyllotoxin derivatives obtained by 4β-N-acetylamino substitution at C-4 position was designed, synthesized, and evaluated for *in vitro* cytotoxicity against four human cancer cell lines (EC-9706, HeLA, T-24 and H460) and a normal human epidermal cell line (HaCaT). The cytotoxicity test indicated that most of the derivatives displayed potent anticancer activities. In particular, compound **12h** showed high activity with IC_50_ values ranging from 1.2 to 22.8 μM, with much better cytotoxic activity than the control drug etoposide (IC_50_: 8.4 to 78.2 μM). Compound **12j** exhibited a promising cytotoxicity and selectivity profile against T24 and HaCaT cell lines with IC_50_ values of 2.7 and 49.1 μM, respectively. Compound **12g** displayed potent cytotoxicity against HeLA and T24 cells with low activity against HaCaT cells. According to the results of fluorescence-activated cell sorting (FACS) analysis, **12g** induced cell cycle arrest in the G2/M phase accompanied by apoptosis in T24 and HeLA cells. Furthermore, the docking studies showed possible interactions between human DNA topoisomerase IIα and 12g. These results suggest that **12g** merits further optimization and development as a new podophyllotoxin-derived lead compound.

## Introduction

Currently, cancer has become one of the most serious threats to public health across the globe, and it is considered the leading cause of death in developed countries and the second leading cause of death in developing countries (Jemal, 2011). Natural products have been an effective and successful method to identify novel hits and leads for curing this deadly disease (Cragg and Newman, 2013; Newman and Cragg, 2016). Podophyllotoxin (PPT, **1**), a natural product extracted from the plants of the *Podophyllum* family, exhibited significant anti-tumor and anti-viral activities, attracting great interest as a hallmark molecule because of its biological activities (MacRae et al., 1989; Lear and Durst, 1996; Gordaliza et al., 2004; Nandagopal and Routh, 2017). It has been stated that PPT exerted antitumor activity via inhibiting microtubule bundle formation in mitosis metaphase, preventing the formation of the spindle and arresting cell division in metaphase (G2/M stage) (Damayanthi and Lown, 1998; Ravelli et al., 2004; Hartley et al., 2012).

**Figure 1:**
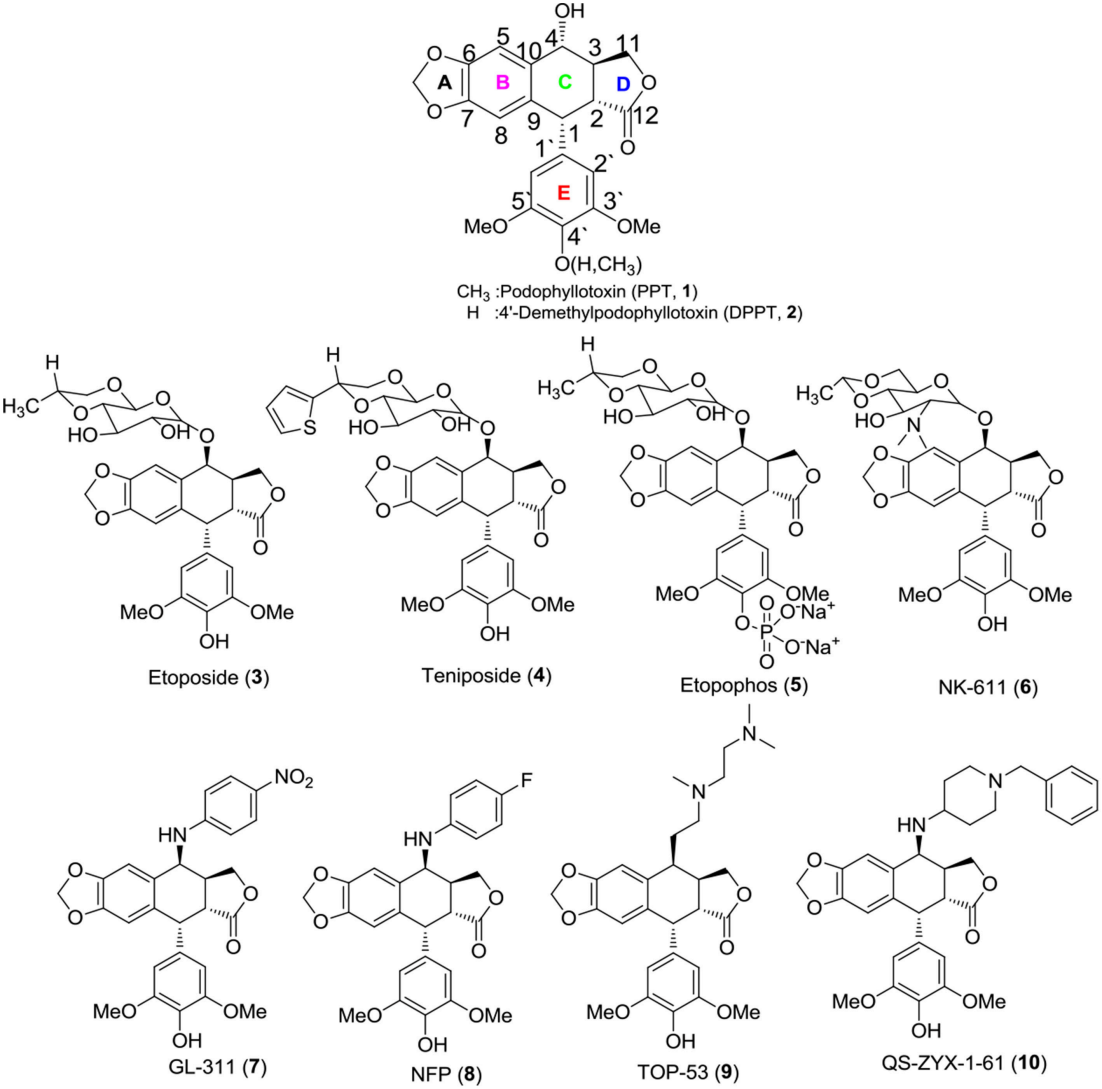
Structures of PPT, DPPT, and related compounds.

Its excellent activity attracted much attention from scientists; however, PPT exerted serious side effects during cancer chemotherapy and had some therapeutic limitations, including high toxicity, poor water solubility, drug resistance and other unfavorable profiles (Damayanthi and Lown, 1998; Canel et al., 2000; Gordaliza et al., 2000). PPT was mainly used to cure dermatosis in clinical practice (Komericki et al., 2011; Lopez-Lopez et al., 2015). Hence, there is a need to develop safe and efficacious PPT derivatives for anticancer therapy to overcome the shortcomings. Aiming to find novel PPT derivatives with high efficiency and low toxicity, researchers carried out a series of structural modifications with podophyllin ingredients and obtained three potent semisynthetic glucoconjugates based on 4′-demethylpodophyllotoxin (DPPT, **2**), including etoposide (**3**), teniposide (**4**), and a water-soluble prodrug of etoposide, named etopophos (etoposide phosphate, **5**) (Figure 1) (Keller-Juslen et al., 1971; Greco and Hainsworth, 1996; Damayanthi and Lown, 1998; Hande, 1998). The semisynthetic glucoconjugates displayed favorable water solubility and are currently in clinical use for the treatment of various malignancies, including small cell lung cancer, testicular carcinoma, lymphoma, non-lymphocytic leukemia, and multiform glioblastoma (Issell, 1982; Loike, 1982; Witterland et al., 1996; Liu et al., 2015; Moon et al., 2017).

Although the solubility problem had been resolved, toxicity issues remain a challenge for the semisynthetic glucoconjugates. Furthermore, some non-sugar substituted PPT derivatives were developed, e.g., NK-611 (**6**), GL-331 (**7**), NPF (**8**), TOP-5 (**9)**, and QS-ZYX-1-61 (**10**), which displayed a better pharmacology profile, exhibited excellent anti-tumor activity and reached clinical trials for the treatment of a broad spectrum of tumors (Utsugi et al., 1996; Shimizu et al., 2002; You, 2005; Lv and Xu, 2011; Kamal et al., 2015; Liu et al., 2015). These novel derivatives were obtained by modifying at the C-4 position of PPT/DPPT. Unlike **1**, the newly developed derivatives exerted high anti-tumor activity by inhibiting DNA topoisomerase II (Damayanthi and Lown, 1998; Gordaliza et al., 2000; Wilstermann et al., 2007; Kamal et al., 2015). Thus, the structural optimization of PPT/DPPT is a desirable way to develop inhibitors of DNA topoisomerase II as new anticancer drugs (Srivastava et al., 2005; Khazir et al., 2014).

According to the previous structure–activity relationship (SAR) between PPT/DPPT and the clinical drug candidates, it has been proved that the tetralin nucleus structure of PPT/DPPT is important to keep the anti-tumor activity, which should remain unchanged; the dioxolane ring was essential. Additionally, the 4′-OCH_3_ moiety was generally not essential, removal of it or introduction of the appropriate moiety at the C-4′ position was acceptable. The C-4 position was one of the most important locations for structural optimization, and 4β-configuration was optimal and 4β-anilino substituted podophyllotoxin derivatives, including GL-331 (7), NPF (8), and QS-ZYX-1-61 (10), all of which were epipodophylotoxin derivatives (4β-podophyllotoxin derivatives), showed potent cytotoxic activity against some human parental and drug-resistant cancer cell lines. The side chain structure, containing one or more basic center (amino group) at the C-4 position of PPT/DPPT, not only kept efficient anti-tumor activity but also reduced toxicity. In addition, the amino group easily turned into salt to improve the water solubility of the PPT/DPPT-derived derivatives (Zhang et al., 2010; Hyder et al., 2015; Kamal et al., 2015; Liu et al., 2015; Yu et al., 2017). GL-331 (7), NPF (8), TOP-53 (9), and QS-ZYX-1-61 (10), bearing a hydrophobic side-chain structure at C-4, exerted modest toxicity and potent anticancer activity. Hence, modification of semisynthetic non-glucoconjugates containing the hydrophobic side chain structure at C-4 is another feasible way to optimize the structure of PPT.

**Figure 2:**
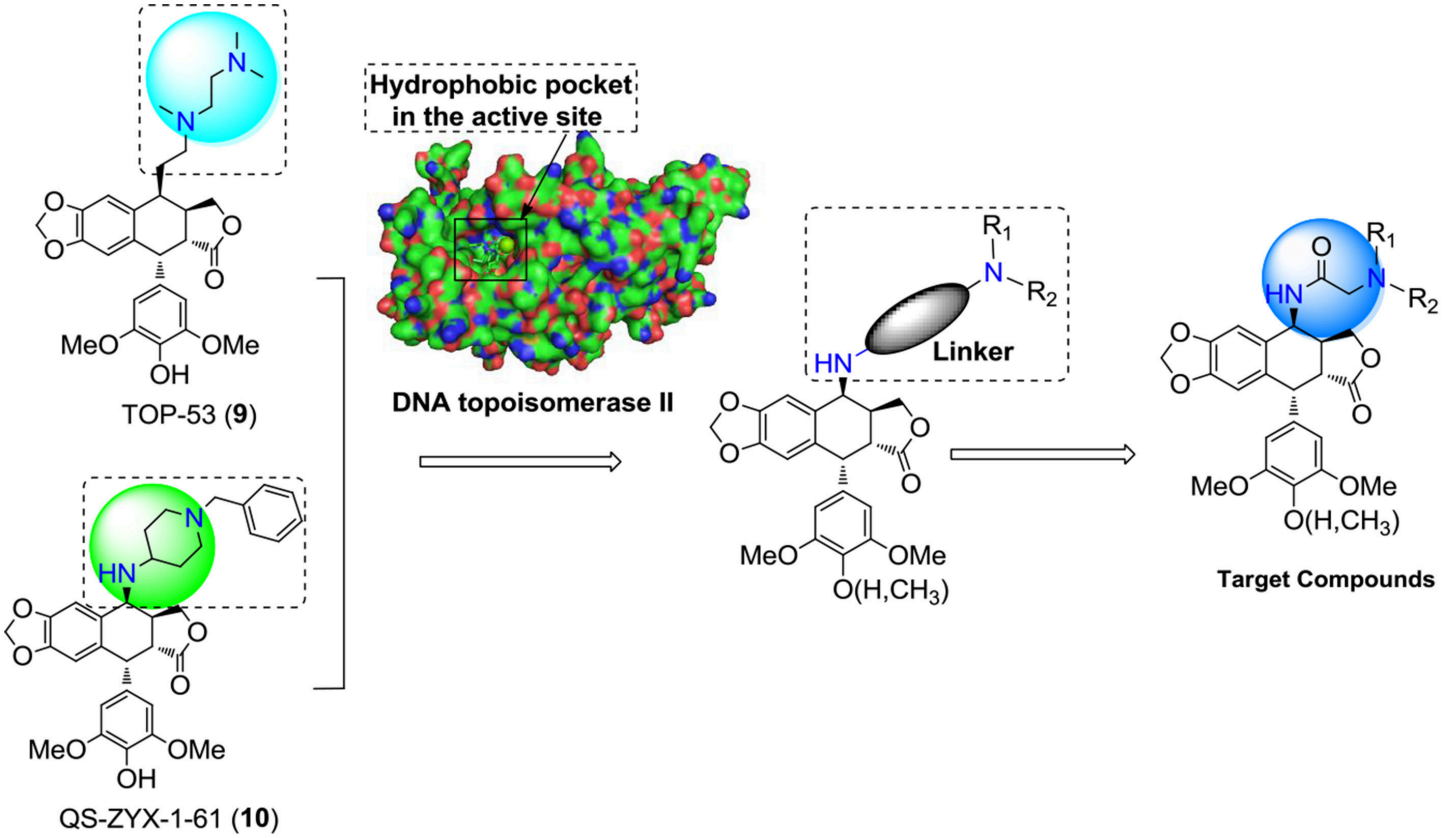
Rational design of the target compounds.

Based on the above analysis and the structures of newly developed clinical candidates **9**-**10** (Figure 2), we thought modification of non-glucoconjugates was another clue for designing new PPT derivatives. Taking into consideration the limitations of PPT derivatives, the target compounds was aimed at increasing the interactions with the target human DNA topoisomerase IIα and simultaneously to overcome the toxicity problems of PPT derivatives. We anticipated that introduction of the hydrophobic side-chain structure at C-4 might increase the interactions with the hydrophobic pocket in the active site of human DNA topoisomerase II. Moreover, we used an N-acetylamino at C4 of PPT as a linker in the side chain. With the amino-group, the designed derivatives were able to undergo a salt-formation process under suitable conditions, which could also improve the required water solubility of PPT drugs. Consequently, an introduction of a side chain containing a diamido group at the C-4 position of PPT/DPPT would be a feasible approach to develop new PPT/DPPT derivatives as anticancer agents.

This paper reported the design and synthesis of a series of PPT/DPPT-derived derivatives (seen in graphical abstract) with the C-4 position of PPT/DPPT coupling 4β-N-acetylamino side chains which contained different types of substituted aliphatic hydrocarbons or aromatic hydrocarbons (types of substituents: aliphatic chain/carbocyclic ring, donating/withdrawing electron groups, steric hindrance groups, and hydrophobic/hydrophilic groups). The cytotoxic activity of the derivatives was tested *in vitro* against four human cancer cell lines (EC-9706, HeLA, T-24, and H460) and a normal human epidermal cell line (HaCaT). Additional biological studies were conducted to analyze how novel compounds of this class affect the cell cycle. Docking studies were performed to investigate the possible binding interactions between synthesized compounds and the human topoisomerase IIα active site and predict the mechanism of action as novel anticancer agents.

**Graphical Abstract:**
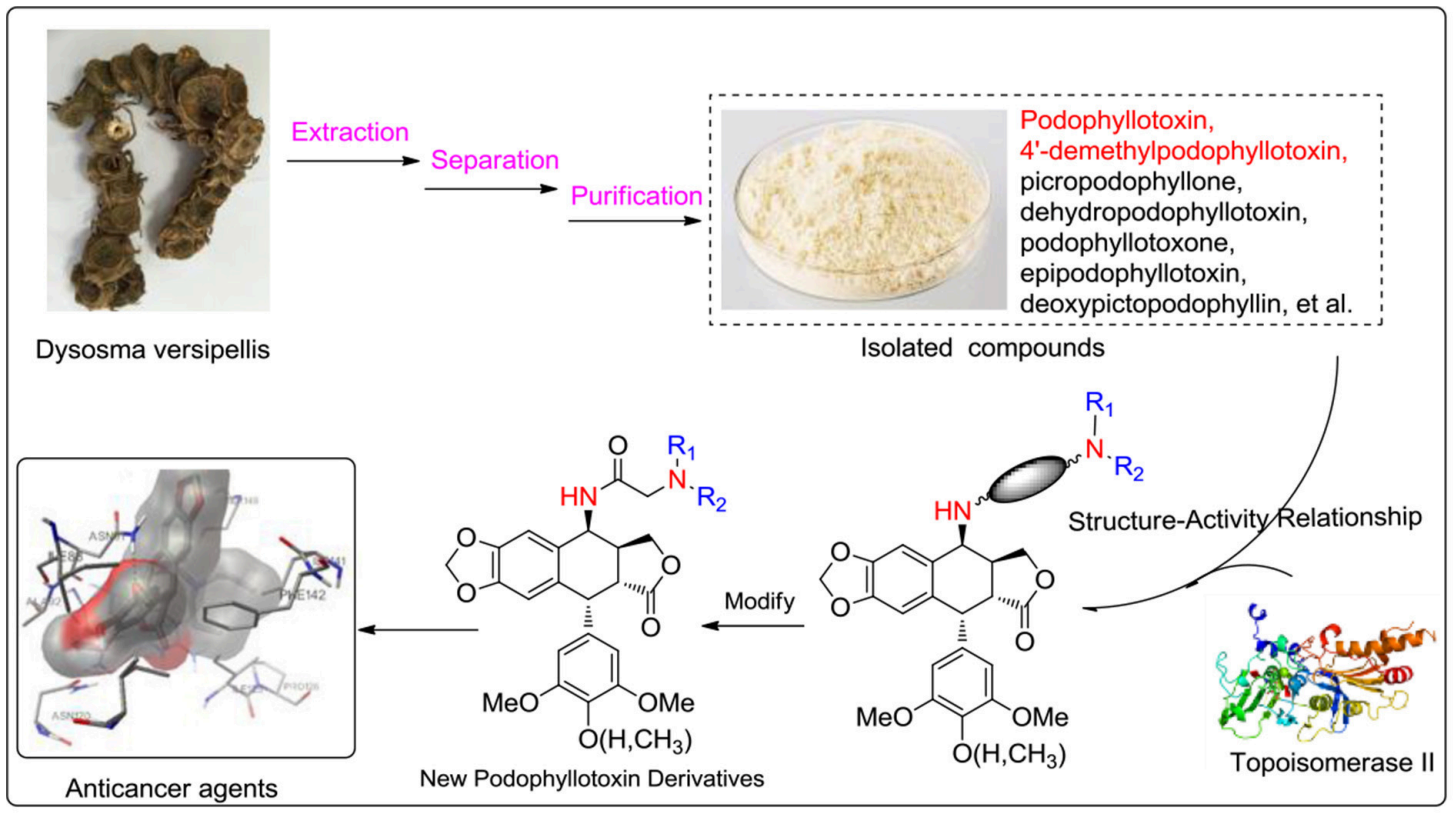
The diagrammatic sketch of our study on podophyllotoxin derivatives.

## Chemistry

**Scheme 1:**
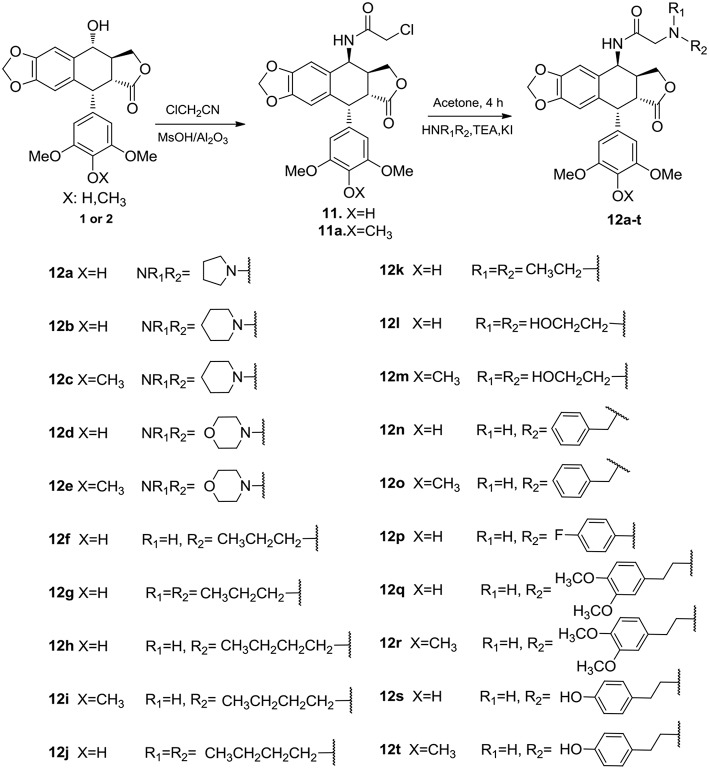
Synthesis of 4β-N-acetylamino substituted PTT and DPPT derivatives **12a-t**.

The synthetic route to 4β-N-acetylamino substituted PPT and DPPT derivatives **12a-t** is illustrated in Scheme 1. The derivatives were prepared with PPT and DPPT as the raw materials. Key intermediates 4β-chloroamido PPT/DPPT (**11** and **11a**) were synthesized in excellent yields by reaction with chloroacetonitrile (ClCH_2_CN) with the presence of 60% w/w methanesulfonate/aluminum oxide (MsOH/Al_2_O_3_). Subsequently, the key intermediate **11** or **11a** was reacted with substituted amines in the presence of potassium carbonate and potassium iodate to afford a series of 4β-N-acetylamino substituted PPT and DPPT derivatives with good yields (**12a-t**). All newly synthesized compounds were purified by column chromatography and their chemical structures were confirmed by ^1^H NMR, ^13^C NMR, and ESI-MS data.

## Biological Evaluation

### Cytotoxicity and SAR

Target compounds **12a–t** were evaluated for *in vitro* cytotoxicity against four human tumor cell lines, including H460 (non-small cell lung carcinoma), HeLA (human cervical carcinoma), EC9706 (human esophageal squamous cell carcinoma), and T24 (human bladder carcinoma), using HaCaT (human immortalized epidermal cells) as a human non-malignant cell line. Etoposide (**3**) was included as a positive control. The screening procedure was based on the 3-(4, 5-dimethylthiazol-2-yl)-2, 5-diphenyltetrazoliun bromide (MTT) growth inhibition assay with triplicate experiments, and the results are summarized in Table 1.

**Table 1:** *In vitro* cytotoxicity of compounds **12a–t** against five human tumor cell lines with etoposide as control (IC_50_).

**Compds**.	**IC**_****50****_ **(μM)**^**a**^				
	**EC9706**	**HeLA**	**T24**	**H460**	**HaCaT**
**12a**	>50	12.72 ± 1.38	>50	9.61 ± 0.22	18.16 ± 1.28
**12b**	>50	5.72 ± 0.74	>50	5.91 ± 0.65	5.17 ± 0.36
**12c**	>50	>50	>50	>50	1.89 ± 0.27
**12d**	>50	>50	>50	>50	>50
**12e**	>50	>50	>50	>50	>50
**12f**	11.65 ± 0.26	23.85 ± 1.04	18.63 ± 1.57	2.54 ± 0.34	2.59 ± 0.13
**12g**	>50	2.34 ± 0.39	2.98 ± 0.26	24.19 ± 0.77	>50
**12h**	22.78 ± 0.32	1.21 ± 0.31	12.10 ± 1.28	1.57 ± 0.37	1.54 ± 0.29
**12i**	>50	>50	>50	37.77 ± 1.01	>50
**12j**	35.56 ± 0.91	42.92 ± 0.69	2.68 ± 0.40	26.12 ± 0.98	49.08 ± 1.67
**12k**	>50	45.76 ± 0.71	>50	>50	>50
**12l**	>50	>50	>50	>50	4.21 ± 0.17
**12m**	>50	36.40 ± 0.49	>50	>50	>50
**12n**	40.13 ± 2.43	6.25 ± 0.21	>50	16.51 ± 0.39	13.30 ± 0.80
**12o**	11.34 ± 0.22	8.55 ± 0.28	>50	26.86 ± 0.92	2.14 ± 0.33
**12p**	>50	>50	>50	9.75 ± 0.26	>50
**12q**	20.68 ± 0.72	4.57 ± 0.30	>50	3.43 ± 0.17	3.69 ± 0.19
**12r**	>50	>50	>50	>50	>50
**12s**	14.32 ± 0.69	2.27 ± 0.26	12.7 ± 0.73	5.94 ± 0.18	8.67 ± 0.72
**12t**	>50	>50	>50	42.07 ± 1.21	>50
Etoposide	>50	3.12 ± 0.35	8.43 ± 0.33	43.17 ± 1.45	48.74 ± 1.69

*Human tumor cells were treated with different concentrations of samples for 48 h (n = 3 independent experiments)*.

a *Data are presented as IC_50_ (μM, the concentration of 50% proliferation inhibitory effect)*.

Notably, in comparison to the data of Table 1, it was clear that DPPT-derived derivatives showed better cytotoxic activity than the PPT-derived derivatives with C-4′ OCH_3_ substituent, suggesting that demethylation at C-4′ of PPT could improve the cytotoxicity against tumor cell lines. Among the analogs derived from DPPT, compounds **12h** and **12s** showed superior activity (IC_50_ 1.21–1.57 and 2.27–5.94 μM, respectively) compared with etoposide (IC_50_ 3.12–43.17 μM) against HeLA and H460 tumor cell lines. Meanwhile, the two compounds also showed significant cytotoxicity against the HaCaT cell line with IC_50_ values of 1.54 and 8.67 μM, respectively.

Compounds **12d-e** containing a morpholine ring substituent at the C-2′′ position of the acetylamino moiety lost their cytotoxicity against the cancer cell lines as well as the normal cell line with IC_50_ values >50 μM. Moreover, Compounds **12a-b** bearing a five or six-membered aliphatic ring displayed poor cytotoxicity, which showed comparable potency to compound **12n** with a phenyl group. When the 2′′-substituent was changed from phenyl (**12n**) to p-hydroxyphenylethyl (**12s**), the cytotoxicity against HeLA improved with IC_50_ values from 6.26 to 2.27 μM. Compound **12k** with disubstituted ethyl group showed poor cytotoxicity, while compound with disubstituted n-propyl group (**12g**) showed more significant improved potency and selectivity of cytotoxicity against the cancer cell lines than the mono-substituted compound (**12f**), which was similar to that observed between **12h** and **12j**. The results suggested that an introduction of the hydrophobic disubstituted alkyl group might be important for the cytotoxicity and selectivity of antitumor effects. To assess the inhibitory effect of the active derivative (**12g**) on cancer cells, Hoechst 33258 staining and flow cytometry analysis on HeLA and T24 cells were conducted.

### Morphological Changes and Apoptosis

**Figure 3:**
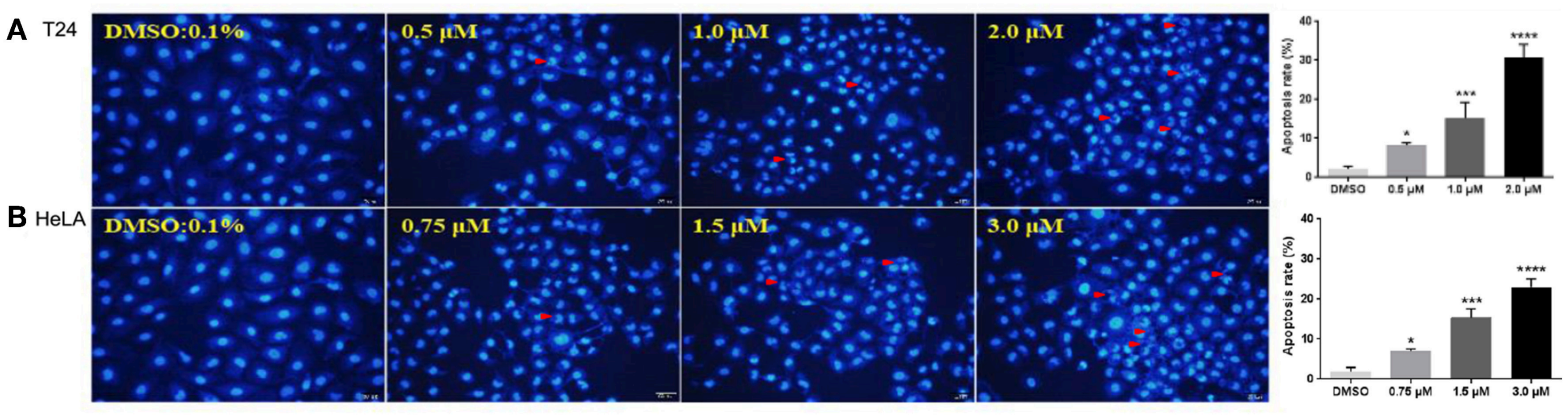
Fluorescent images of Hoechst 33258 staining showing compound **12g** induced cell death with 0.1% DMSO vehicle as the control. Cells were examined under a fluorescence microscope. The arrows indicated the formation of apoptotic bodies with condensed chromatin or fragmented chromatin. **(A)** Treatment of T24 cells with **12g** for 24 h. **(B)** Treatment of HeLA cells with **12g** for 24 h. The apoptosis rates of T24 and HeLA cells with treatment of **12g** were quantify and analyzed by One-way ANOVA (*n* = 3 in each treatment). ^*^P< 0.05, ^***^P < 0.001, and ^****^P < 0.0001 compared with the vehicle (0.1% DMSO). The statistical data are presented as mean ± S.D. Scale bar = 50 μm.

Apoptosis is one of the major pathways leading to the process of cell death. Visualization of chromatin condensation as well as nuclear shrinking and fragmentation—known as classic characteristics of apoptosis—was carried out in the presence of a representative compound **12g** by staining T24 and HeLA cells with Hoechst 33258, by which apoptosis was confirmed as the cause of reduced cell viability. As shown in Figure 3, treatment of **12g** at three different gradient concentrations markedly increased chromatin condensation, nuclear fragmentation and morphological changes as compared to the vehicle (0.1% DMSO)-treated cells, demonstrating that the cells undergo apoptosis (the arrowhead indicated an apoptotic nucleus), while negative control cells displayed excellent growth characteristics. Thus, these results evidently indicated that the compound **12g** is effective in inducing cellular apoptosis.

### Cell Cycle Analysis

**Figure 4:**
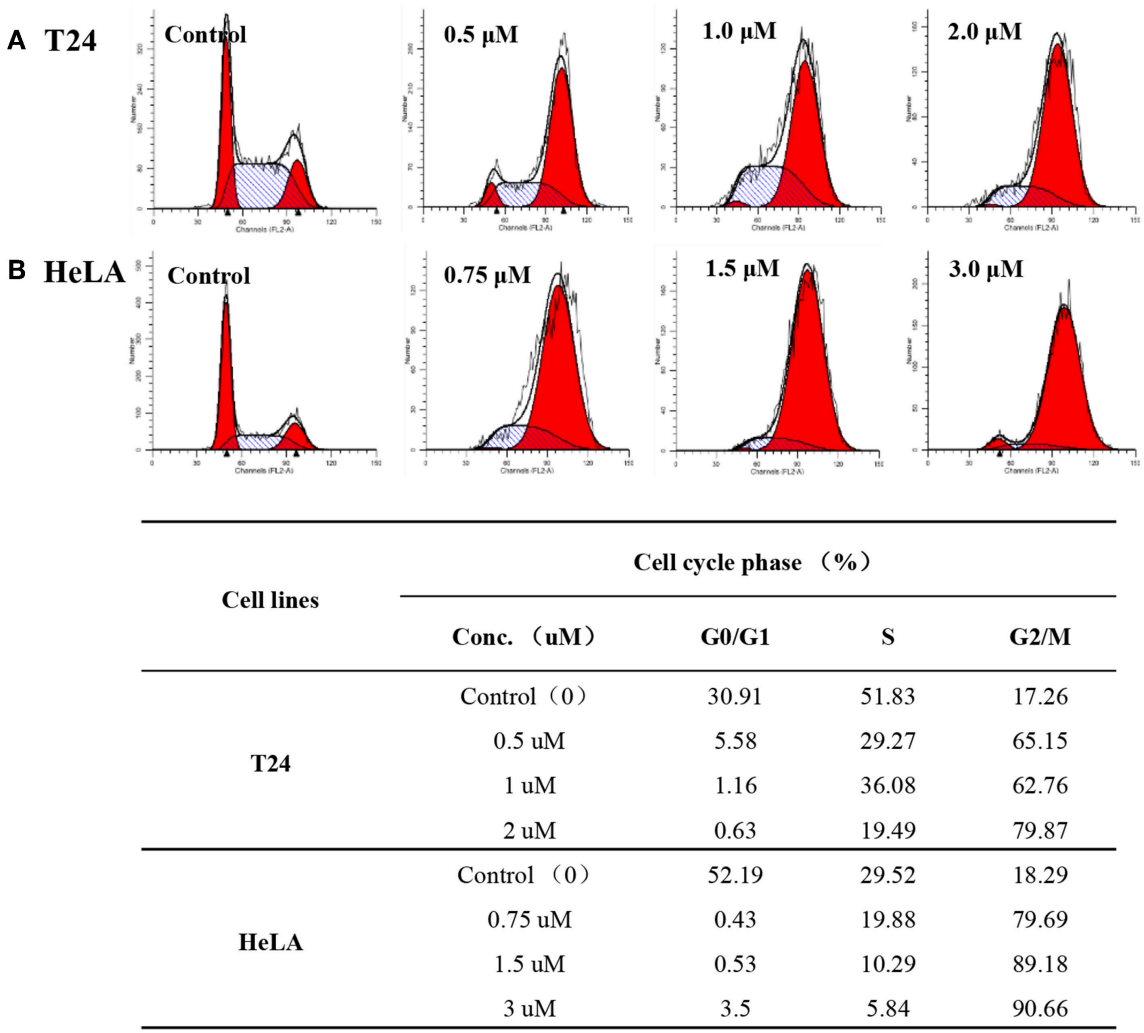
Compound **12g** affected the cancer cell cycle distribution at three different gradient concentrations with 0.1% DMSO vehicle as the control. **(A)** T24 cells treated with **12g** for 24 h. **(B)** HeLA cells treated with **12g** for 24 h.

Flow cytometry analysis was carried out to evaluate cell cycle changes and gain further insight into the mode of action with respect to the anti-proliferative effect of compound **12g**. HeLA and T24 cancer cells were treated with the selected compound **12g** for 48 h at three different gradient concentrations. The cell cycle accumulation was examined by the propidium iodide staining and cytometric quantification after the cancer cells were cultured for 24 h. As shown in Figure 4, in HeLA cells treated with **12g**, 79.69, 89.18, and 90.66%, respectively, of cells were in G2/M phase, compared to only 18.29% of control (untreated) cells in this phase. It was clear that the percentage of G2/M cells showed a remarkable increase in HeLA cells along with the increasing concentrations of **12g**. Additionally, treated compound **12g** with concentrations of 0.75, 1.5, and 3.0 μM resulted in large accumulations of T24 cells at G2/M phase of 65.15, 62.76, and 79.87%, respectively. These results indicated that compound **12g** efficiently arrested the cancer cells at G2/M phase.

## Docking Study

**Figure 5:**
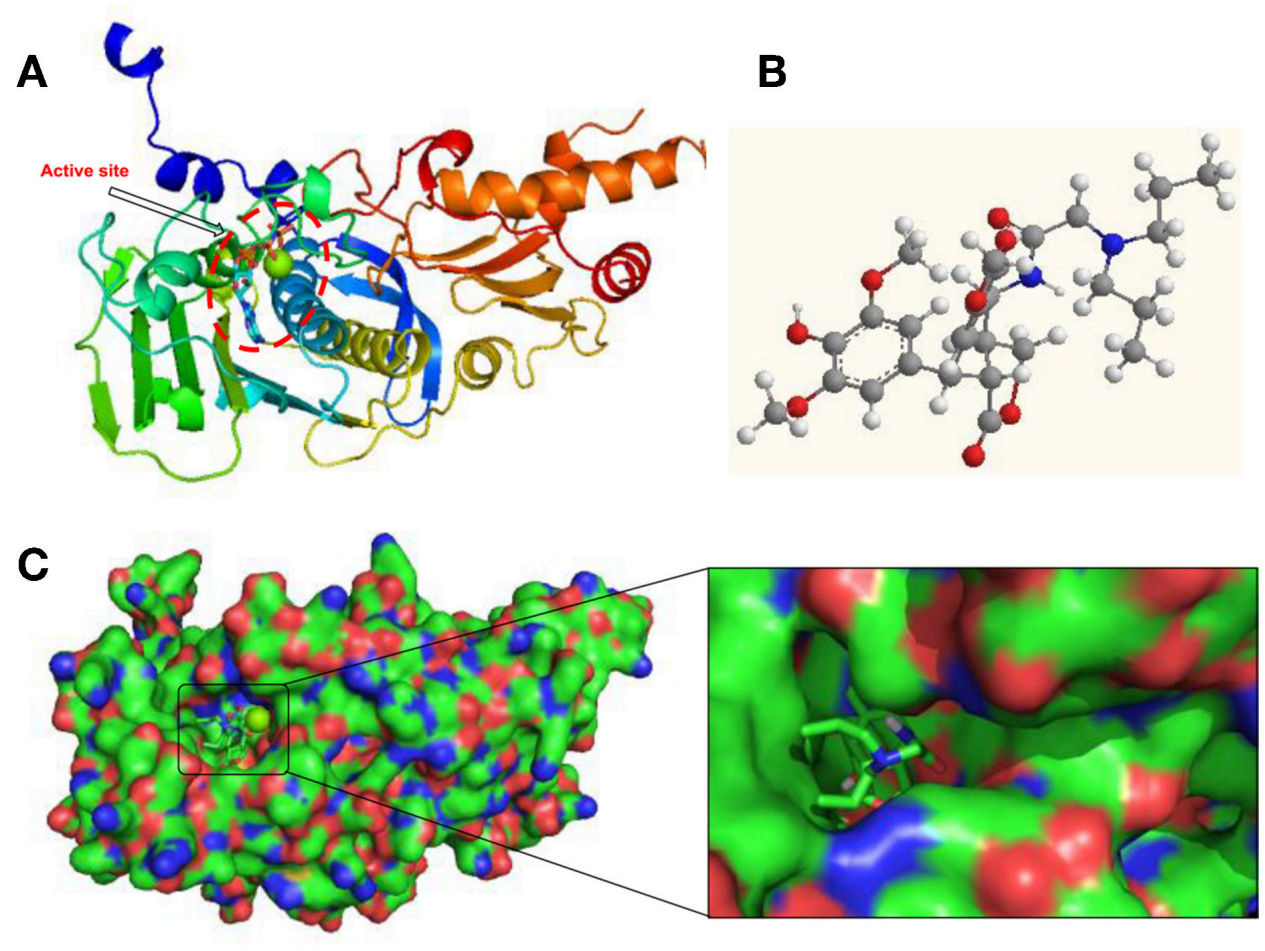
Human DNA topoisomerase IIα active site in complex with its ligand AMP-PNP or synthesized compound **12g**. **(A)** X-ray crystal structure of human DNA topoisomerase IIα active site in complex with AMP-PNP. **(B)** The 3D chemical structure of compound **12g**. **(C)** The surface structure of human DNA topoisomerase IIα binding pocket in complex with **12g**.

Aiming to investigate the possible binding interactions of synthesized compound **12g** inside the human topoisomerase IIα active site and to predict the mechanism of action as anti-cancer agent, a molecular docking study was performed using Autodock 4.0 as modeling software. An X-ray crystal structure of the human DNA topoisomerase IIα active site in complex with its ligand AMP-PNP was downloaded from the protein data bank (PDB code: 1ZXM). As shown in Figure 5A, the three-dimensional structure of the 1ZXM crystal is composed of 7 α-helixes, 13 β-folds, and random coils, which subsequently forms a hydrophobic groove functioning as the active site or binding pocket (Figures 5A,B). The active site is located at the center of the enzyme, where small molecule compounds including podophyllotoxin derivatives occupy to block the entry of ligands into the binding site (Figure 5C).

**Figure 6:**
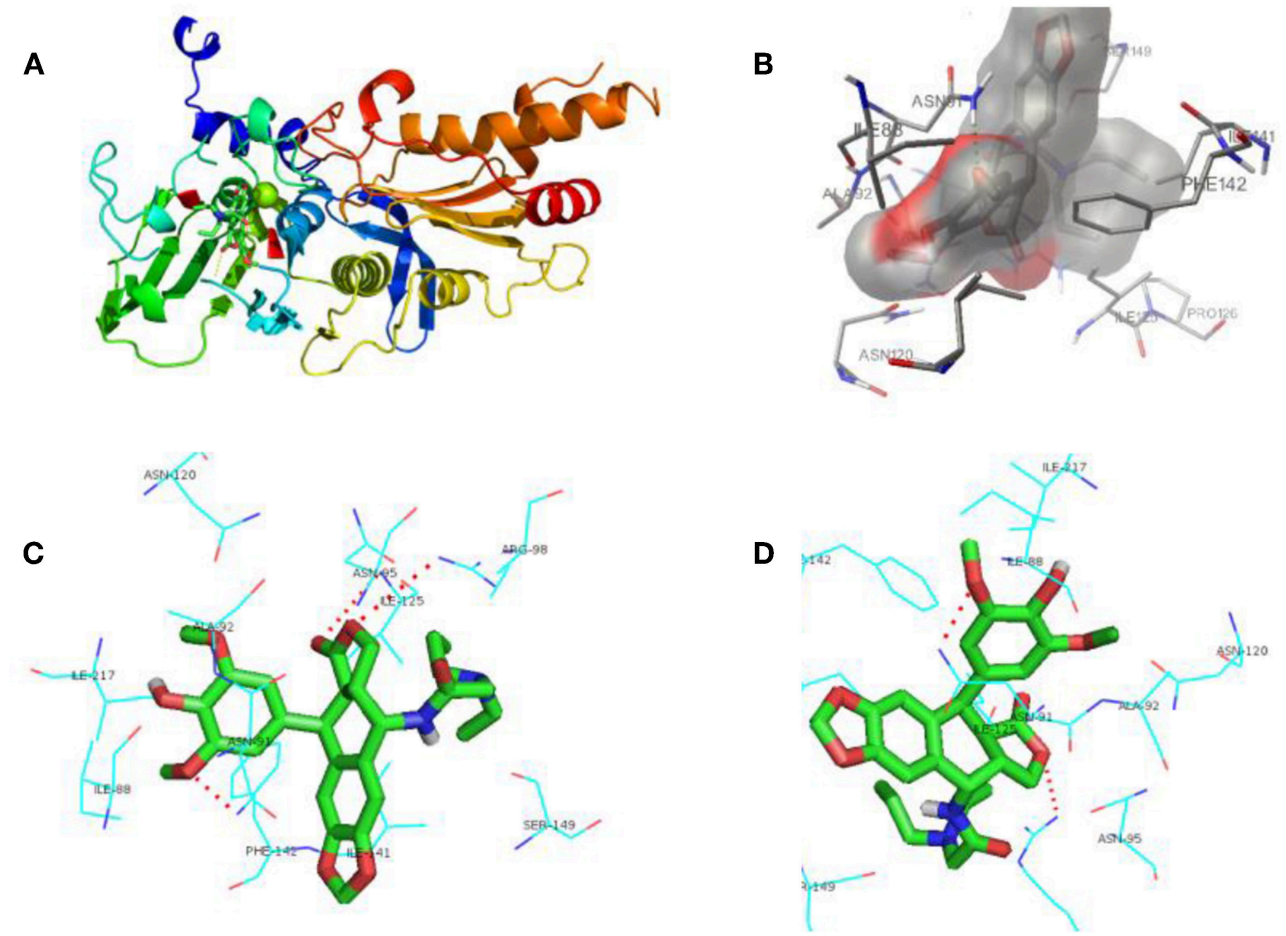
Docking poses for compound **12g** in the active site of human DNA topoisomerase IIα. **(A)** X-ray crystal structure of human DNA topoisomerase IIα active site in complex with **12g** (green). **(B)** Inhibitor **12g** was bound to the hydrophobic groove of DNA topoisomerase IIα. **(C,D)** View of compound **12g** (green) docked in the binding residues of human topoisomerase IIα from different perspectives.

In Figure 6, it represented the docking pose of **12g** in the binding pocket, where it is enclosed by ILE88, ASN91, ALA92, ASN95, ARG98, ASN120, ILE125, ILE141, PHE142, SER149, and ILE217 residues. Inhibitor **12g** was found to form 3 hydrogen bonding interactions with ASN91, ARG98 and ILE125 amino acid residues inside the DNA topoisomerase IIα active site with distances of 2.9, 3.4, and 3.5 Å, respectively, and the following binding energy: E-score = −10.46 Kcal/mol. The aromatic ring at the C-1 position of DPPT was involved in π-π stacking with the aromatic residue of PHE142. Additionally, hydrophobic interactions of dipropyl with ILE125 and ILE141 served to stabilize the side-chain structure of **12g**, accounting for its good antitumor activity in MTT testing. As shown in MTT testing, whereas compound **12i** with a butyl side chain was not more cytotoxic than **12g**, we thought that dibutyl side chain of 12i was more flexible that n-propyl group of **12g**, which might weaken the hydrophobic interaction with human topoisomerase II hydrophobic pocket. In addition, a bigger group occupied a larger space in the receptor pocket, leading to the necessity for more hydrophobic amino acid residues to maintain conformation stability. As a result, bigger side chains would probably be positioned outside the active pocket.

In accordance with the data of MTT testing *in vitro*, docking results also showed that PPT derivative **12g**, which contained the hydrophobic side-chain structure, exerted relatively potent cytotoxicity on cancer cells compared with other analogs, which proved our expectations of the design. Hence, an introduction of hydrophobic side-chain structure with appropriate size at the C-4 position of DPPT may increase the biological activity via interaction with the hydrophobic groove of DNA topoisomerase IIα. All these interactions enhanced the hydrophobic groove binding affinity of the 4β-N-acetylamino substituted podophyllotoxin derivatives.

## Conclusions

According to previous SAR studies on PPT/DPPT and their clinical drug candidates, a series of novel PPT/DPPT derivatives was designed and synthesized to increase the interactions with the target human DNA topoisomerase IIα and simultaneously to improve the toxicity issues of DPPT derivatives. These derivatives were evaluated for anti-tumor activity *in vitro* against several human tumor cell lines using an MTT assay. Among the analogs, compounds **12h** and **12s** showed superior activity against HeLA and H460 tumor cell lines with IC_50_ values of 1.21/1.57 and 2.27/5.94 μM, respectively. Moreover, compound **12g** derived from DPPT was one of the most promising synthetic derivatives, with greater potency and selectivity of cytotoxicity than the positive control etoposide, and was selected as lead molecule for further development, inducing cell cycle arrest in the G2/M phase and apoptosis in both HeLA and T24 cells. The above preliminary investigation of cytotoxicity and SAR suggested that a substituted hydrophobic group at C-4 of PPT and free C-4′ OH had a major impact on the cellular activity. An introduction of hydrophobic disubstituted alkyl group on the acetylamino moiety was advantageous. Removal of CH3 at the C-4′ position of PPT would increase water solubility, which contributed to enhancing bioavailability. Introducing polar groups that were hydrolyzable *in vivo* via enzymes or non-enzymes at the C-4′ OH position of DPPT was a feasible way to achieve a DPPT prodrug like etopophos. The docking studies disclosed hydrophobic side-chain structure with appropriate size at the C-4 position of DPPT would enhance the hydrophobic interactions with the hydrophobic amino acid residues within human DNA topoisomerase IIα. In conclusion, due to **12g** and other derivatives′ excellent anti-proliferative potency and remarkable apoptosis-inducing activity, further studies to substantiate and improve activity profiles are ongoing.

## Experimental Section

### Chemistry

All solvents, reagents, and chemicals for the synthesis of the compounds were of analytical grade, purchased from commercial sources and used without further purification, unless otherwise specified. Melting points were taken on a Kofler melting point apparatus and are uncorrected. ^1^H NMR and ^13^C NMR spectra were measured on a Bruker Ascend^TM^-400 spectrometer (Bruker Company, USA) with tetramethylsilane (TMS) as an internal standard. All chemical shift values are expressed in d parts per million. Mass spectra were recorded on a Waters-XEVO UPLC/MS/MS spectrometer with ESI source as ionization. Podophyllotoxin (PPT, **1**) and 4′-demethylpodophyllotoxin (DPPT, **2**) were isolated from the Chinese medicinal herb *Dysosma versipellis* and served as the starting materials for the preparation of all new derivatives.

### Chemistry

#### General Synthetic Procedure for the Key Intermediates 4β-Chloroamido-Podophyllotoxin (11) and 4β-Chloroamido-4′- Demethylpodophyllotoxin (11a)

To a stirred mixture of PPT or DPPT (4 mmol) and ClCH_2_CN (10 mL), a homogeneous mixture of MsOH/Al_2_O_3_ (60 mass %, 1 g) was added, and the mixture was irradiated by an ultrasonic generator in a water bath at 60°C for 30 min, then evaporated under reduced pressure (Li et al., 2013). The residue was purified by chromatography on silica gel using EtOAc–petroleum ether to give the key intermediate **11** or **11a**.

#### General Synthetic Procedure for Compounds 12a–t

The key intermediate **11** or **11a** (1.0 mmol) was added to a solution of various substituted amines (1.2 mmol), potassium iodate (0.1 mmol) and potassium carbonate (2.4 mmol) in dry acetonitrile (10 mL). The reaction mixture was stirred for 2.5 h at 65°C, then evaporated under reduced pressure. The residue was purified by chromatography on silica gel using EtOAc–petroleum ether to give compounds **12a–t** (detailed steps and NMR data were available in Supporting Information).

##### 4β-N-[(2′′-(pyrrolidine)-acetamide]-4′-demethyl podophyllotoxin (12a)

Yield 76% from **11** as a white solid; mp: 113-116 °C; ^1^H NMR (400 MHz, DMSO-*d*_6_): δ 8.25 (d, *J* = 9.3 Hz, 1H, CONH), 6.97 (s, 1H, H-5), 6.83 (s, 1H, H-8), 6.44 (s, 2H, H-2′, 6′), 6.02 (2s, 2H, OCH_2_O), 5.14 (dd, *J* = 9.1, 6.2 Hz, 1H, H-4), 4.39 (s, 1H, OH-4′), 4.28 (m, 1H, H-1), 4.11 (m, 1H, 11β-H), 3.80 (m, 1H, 11α-H), 3.71 (s, 6H, 3′,5′-OCH_3_), 3.64 (m, 1H, H-2), 3.34 (m, 1H, H-3), 3.20 (d, 2H, COCH_2_), 2.55 (m, 4H, CH_2_NCH_2_), 1.71 (m, 4H, CH_2_CH_2_); ^13^C NMR (101 MHz, DMSO-*d*_6_): δ 179.13, 172.60, 170.51, 148.35, 146.93, 146.80, 134.73, 131.67, 131.02, 129.61, 110.10, 106.09, 105.47, 101.48, 68.18, 58.86, 56.49, 54.09, 46.36, 45.63, 44.08, 37.70, 23.81, 21.61; ESI-MS: m/z 511.5 [M+H]^+^.

##### 4β-N-[2′′-(piperidine)-acetamide)]-4′-demethylpodophyllotoxin (12b)

Yield 81% from **11** as a white solid; mp: 107-110 °C; ^1^H NMR (400 MHz, CDCl_3_): δ 8.30 (d, *J* = 8.6 Hz, 1H, CONH), 8.02 (s, 1H, OH-4′), 6.80 (s, 1H, H-5), 6.65 (s, 1H, H-8), 6.38 (s, 2H, H-2′, 6′), 5.98 and 5.96 (2s, *J* = 8.1 Hz, 2H, OCH_2_O), 5.31 (s, 1H, H-4), 4.47 (d, 1H, H-1), 4.40–4.30 (m, 1H, 11β-H), 4.15 (m, 1H, 11α-H), 3.83 (s, 6H, 3′,5′-OCH_3_), 3.47 (dd, *J* = 10.5, 2.9 Hz, 1H, H-2), 3.35 (d, 2H, COCH_2_), 3.30(m, 1H, H-3), 2.74 (t, 4H, CH_2_NCH_2_), 1.72 (m, 4H, CH_2_), 1.52 (m, 2H, CH_2_); ^13^C NMR (101 MHz, CDCl_3_): δ 178.61, 176.01, 168.97, 147.57, 147.40, 147.31, 133.73, 132.60, 130.59, 128.38, 110.10, 106.04, 104.55, 101.33, 68.45, 60.83, 56.44, 54.50, 47.79, 45.43, 45.32, 38.06, 25.06, 22.91, 21.24; ESI-MS: m/z 525.5 [M+H]^+^, 563.6 [M+K]^+^.

##### 4β-N-[2′′-(piperidine)-acetamide)]-podophyllotoxin (12c)

Yield 87% from **11** as a white solid; mp: 229-231 °C; ^1^H NMR (400 MHz, CDCl_3_): δ 7.66 (d, *J* = 7.5 Hz, 1H, CONH), 6.71 (s, 1H, H-5), 6.55 (s, 1H, H-8), 6.30 (s, 2H, H-2′, 6′), 5.99 (s, 2H, OCH_2_O), 5.44 (s, 1H, H-1), 5.21 (dd, *J* = 7.5, 4.4 Hz, 1H, H-4), 4.63 (d, *J* = 4.7 Hz, 1H, 11β-H), 4.43 (m, 11α-H), 3.81 (s, 3H, 4′-OCH_3_), 3.75 (s, 6H, 3′,5′-OCH_3_), 3.11 (d, 2H, COCH_2_), 3.04 (m, 1H, H-2), 2.92 (d, 1H, H-3), 2.55 (d, 4H, CH_2_NCH_2_), 1.67 – 1.51 (m, 4H, CH_2_CH_2_), 1.50 – 1.41 (m, 2H, CH_2_); ^13^C NMR (101 MHz, CDCl_3_): δ 174.40, 170.47, 152.62, 148.33, 147.66, 137.21, 134.82, 132.27, 129.03, 110.19, 108.69, 108.17, 101.62, 69.02, 61.67, 60.77, 56.22, 54.84, 47.69, 43.82, 41.81, 37.30, 25.77, 25.41, 23.29, 21.30; ESI-MS: m/z 561.6 [M+Na]^+^.

##### 4β-N-[2′′-(morpholino)-acetamide]-4′-demethylpodophyllotoxin (12d)

Yield 82% from **11** as a white solid; mp: 109–111°C; ^1^H NMR (400 MHz, DMSO-*d*_6_): δ 8.33 (s, 1H, OH-4′**)**, 8.26 (d, *J* = 8.2 Hz, 1H, CONH), 6.96 (s, 1H, H-5), 6.85 (s, 1H, H-8), 6.45 (s, 2H, H-2′, 6′), 6.03 (s, 2H, OCH_2_O), 5.14 (s, 1H, H-4), 4.39 (s, 1H, H-1), 4.28 (m, 1H, 11β-H), 4.12 (m, 1H, 11α-H), 3.81 (m, 1H, H-2), 3.71 (s, 6H, 3′,5′-OCH_3_), 3.61 (s, 4H, CH_2_OCH_2_), 3.45 (s, 1H, H-3), 3.37 (s, 4H, CH_2_NCH_2_), 3.10 (s, 2H, COCH_2_); ^13^C NMR (101 MHz, DMSO-*d*_6_): δ 179.11, 169.83, 148.36, 146.93, 146.84, 134.75, 131.73, 131.04, 129.52, 115.65, 115.44, 115.08, 115.00, 110.10, 106.16, 105.51, 101.50, 68.14, 66.54, 61.54, 56.50, 53.57, 46.41, 45.58, 44.03, 37.74, 19.03; ESI-MS: m/z 527.5 [M+H]^+^.

##### 4β-N-[(2′′-morpholino)-acetamide]-podophyllotoxin (12e)

Yield 81% from **11** as a white solid; mp: 119-121 °C; ^1^H NMR (400 MHz, CDCl_3_): δ 7.43 (d, *J* = 9.1 Hz, 1H, CONH), 6.73 (s, 1H, H-5), 6.55 (s, 1H, H-8), 6.39 (s, 2H, H-2′, 6′), 5.97 and 5.96 (2s, 2H, OCH_2_O), 5.39 (dd, *J* = 9.1, 5.1 Hz, 1H, H-4), 4.37–4.31 (m, 2H, H-11), 3.84 (s, 3H, 4′-OCH_3_), 3.82 (s, 6H, 3′,5′-OCH_3_), 3.75 (s, 1H, H-1), 3.72 (t, 4H, morpholino-CH_2_OCH_2_-), 3.34 (dd, *J* = 10.0, 3.8 Hz, 1H, H-2), 3.23 (m, 1H, H-3), 3.06 (d, 2H, COCH_2_), 2.55(m, 4H, morpholino-CH_2_NCH_2_-); ^13^C NMR (101 MHz, CDCl_3_): δ 178.33, 170.42, 153.59, 147.89, 147.25, 138.51, 137.01, 130.72, 128.43, 109.91, 106.75, 105.11, 101.41, 68.72, 67.05, 61.84, 60.88, 56.24, 53.90, 48.00, 45.08, 44.70, 38.26; ESI-MS: m/z 541.6 [M+H]^+^.

##### 4β-N-[2′′-(n-propylamine)-acetamide)]-4′-demethylpodophyllotoxin (12f)

Yield 81% from **11** as a white solid; mp: 199-202 °C; ^1^H NMR (400 MHz, CDCl_3_): δ 7.99(s, 1H, OH-4′) 7.61 (d, *J* = 7.9 Hz, 1H, CONH), 6.73 (s, 1H, H-5), 6.53 (s, 1H, H-8), 6.30 (s, 2H, H-2′, 6′), 5.98 and 5.97 (2s, 2H, OCH_2_O), 5.24 (dd, *J* = 7.9, 4.6 Hz, 1H, H-4), 4.59 (d, *J* = 4.8 Hz, 1H, H-1), 4.44 – 4.35 (m, 1H, 11β-H), 3.83 (m, 1H, 11α-H), 3.76 (s, 6H, 3′,5′-OCH_3_), 3.71 (d, *J* = 7.0 Hz, 1H, H-2), 3.36 (d, 2H, COCH_2_), 2.96 (s, 1H, NH), 2.88 (m, 1H, H-3), 1.54–1.38 (m, 2H, CH_2_), 1.23 (t, *J* = 7.0 Hz, 2H, N-CH_2_-), 0.87 (t, *J* = 7.4 Hz, 3H, CH_3_); ^13^C NMR (101 MHz, CDCl_3_): δ 174.51, 171.84, 148.30, 147.57, 146.53, 134.08, 132.43, 130.29, 129.04, 110.12, 108.90, 107.83, 101.58, 69.00, 58.37, 56.40, 52.03, 47.54, 43.59, 41.88, 37.18, 22.89, 18.39, 11.52; ESI-MS: m/z 499.5 [M+H]^+^.

##### 4β-N-[(2′′-(dipropylamine)-acetamide]-4′-demethylpodophyllotoxin(12g)

Yield 86% from **11** as a white solid; mp: 125-128 °C; ^1^H NMR (400 MHz, DMSO-*d*_6_): δ 8.29 (s, 1H, CONH), 8.12 (s, 1H, OH-4′), 6.77 (s, 1H, H-5), 6.55 (s, 1H, H-8), 6.25 (s, 2H, H-2′, 6′), 6.01 and 5.99 (2s, 2H, OCH_2_O), 5.19 (dd, *J* = 8.4, 4.8 Hz, 1H, H-4), 4.51 (d, *J* = 5.2 Hz, 1H, H-1), 4.31 (t, *J* = 8.0 Hz, 1H, 11β-H), 3.75 (m, 1H, 11α-H), 3.64 (s, 6H, 3′,5′-OCH_3_), 3.37 (s, 2H, COCH_2_), 3.20 (dd, *J* = 14.5, 5.2 Hz, 1H, H-2), 3.03–2.92 (m, 1H, H-3), 2.53 (s, 4H, 2NCH_2_), 1.50–1.35 (m, 4H, 2CH_2_), 0.80 (t, *J* = 7.3 Hz, 6H); ^13^C NMR (101 MHz, DMSO-*d*_6_): δ 174.94, 172.50, 162.78, 147.70, 147.61, 147.05, 135.15, 132.83, 130.56, 130.48, 109.97, 109.32, 108.88, 101.73, 68.81, 56.59, 56.44, 47.16, 43.34, 41.32, 37.03, 36.25, 31.23, 21.53, 11.95; ESI-MS: m/z 541.6 [M+H]^+^.

##### 4β-N-[(2′′-(n-butylamine)-acetamide]-4′-demethylpodophyllotoxin(12h)

Yield 83% from **11** as a white solid; mp: 115-118 °C; ^1^H NMR (400 MHz, DMSO-*d*_6_): δ 8.28 (d, *J* = 8.3 Hz, 1H, CONH), 7.70 (m, 1H, OH-4′), 6.77 (s, 1H, H-5), 6.55 (s, 1H, H-8), 6.25 (s, 2H, H-2′, 6′), 6.02 and 5.99 (2s, 1H, OCH_2_O), 5.21 (dd, *J* = 8.0, 4.7 Hz, 1H, H-4), 4.51 (d, *J* = 5.1 Hz, 1H, H-1), 4.29 (t, 1H, 11β-H), 3.78 (m, 1H, 11α-H), 3.73 (s, 1H, NH), 3.64 (s, 6H, 3′,5′-OCH_3_), 3.26 (d, 2H, COCH_2_), 3.23–3.14 (m, 1H, H-2), 3.03–2.91 (m, 1H, H-3), 2.55 (m, 2H, NCH_2_), 1.44–1.34 (m, 2H, CH_2_), 1.32–0.23 (m, 2H, CH_2_), 0.86 (t, *J* = 7.3 Hz, 3H, CH_3_); ^13^C NMR (101 MHz, DMSO-*d*_6_): δ 174.95, 172.63, 170.82, 147.70, 147.60, 147.03, 135.13, 132.79, 130.60, 130.57, 109.92, 109.42, 108.86, 101.72, 56.42, 51.73, 48.78, 47.16, 43.35, 41.32, 37.02, 31.33, 21.68, 20.26, 14.27; ESI-MS: m/z 535.6 [M+Na]^+^.

##### 4β-N-[(2′′-(n-butylamine)-acetamide]-podophyllotoxin(12i)

Yield 82% from **11** as a white solid; mp: 92-95 °C; ^1^H NMR (400 MHz, DMSO-*d*_6_): δ 8.42 (d, *J* = 8.2 Hz, 1H, CONH), 6.79 (s, 1H, H-5), 6.56 (s, 1H, H-8), 6.30 (s, 2H, H-2′, 6′), 6.02 and 6.00 (2s, 2H, OCH_2_O), 5.23 (dd, *J* = 8.0, 4.6 Hz, 1H, H-4), 4.57 (d, *J* = 5.3 Hz, 1H, H-1), 4.31 (m, 1H, 11β-H), 3.88–3.77 (m, 1H, 11α-H), 3.73 (s, 1H, NH), 3.66 (s, 6H, 3′,5′-OCH_3_), 3.62 (s, 3H, 4′-OCH_3_), 3.34 (s, 2H, COCH_2_), 3.25 (dd, *J* = 14.5, 5.4 Hz, 1H, H-2), 3.05 – 2.94 (m, 1H, H-3), 2.60 (t, *J* = 6.9 Hz, 2H, NCH_2_), 1.49– 1.38 (m, 2H, CH_2_), 1.36–1.21 (m, 2H, CH_2_), 0.86 (t, *J* = 7.3 Hz, 3H, CH_3_); ^13^C NMR (101 MHz, DMSO-*d*_6_): δ 174.86, 172.55, 152.46, 147.78, 147.13, 136.79, 136.21, 132.40, 130.58, 109.91, 109.51, 108.56, 101.77, 60.35, 56.21, 51.15, 48.52, 47.18, 43.53, 41.16, 37.05, 30.77, 21.59, 20.18, 14.22; ESI-MS: m/z 527.6 [M+H]^+^.

##### 4β-N-[(2′′-(dipropylamine)-acetamide]-4′-demethylpodophyllotoxin (12j)

Yield 78% from **11** as a white solid; mp: 70-72 °C; ^1^H NMR (400 MHz, DMSO-*d*_6_): δ 8.03 (d, *J* = 9.3 Hz, 1H, CONH), 6.91 (s, 1H, H-5), 6.79 (s, 1H, OH-4′), 6.56 (s, 1H, H-8), 6.45 (s, 2H, H-2′, 6′), 6.02 (s, 1H, OCH_2_O), 5.15 (dd, *J* = 8.9, 2.7 Hz, 1H, H-4), 4.35 (d, 1H, H-1), 4.09 (m, 1H, 11β-H), 3.80 (m, 1H, 11α-H), 3.71 (s, 6H, 3′,5′-OCH_3_), 3.32 (m, 1H, H-3), 3.18 (dd, *J* = 14.1, 5.4 Hz, 1H, H-2), 3.10 (s, 2H, COCH_2_), 2.45 (m, 4H, CH_2_NCH_2_), 1.39 (m, 4H, 2CH_2_), 1.27 (m, 6H, 2CH_3_). ^13^C NMR (101 MHz, DMSO-*d*_6_): δ 179.01, 171.44, 148.39, 147.61, 146.97, 146.94, 134.82, 131.77, 131.22, 129.58, 108.88, 105.59, 101.54, 57.89, 56.48, 56.43, 54.89, 54.53, 48.97, 45.48, 44.03, 37.83, 31.24, 29.58, 20.58, 20.52, 14.39; ESI-MS: m/z 591.7 [M+Na]^+^.

##### 4β-N-[(2′′-(diethylamine)-acetamide]-4′-demethylpodophyllotoxin (12k)

Yield 84% from **11** as a white solid; mp: 106-108 °C; ^1^H NMR (400 MHz, DMSO-*d*_6_): δ 8.34 (s, 1H, OH-4′), 8.21 (d, *J* = 9.1 Hz, 1H, CONH), 6.96 (s, 1H, H-5), 6.80 (s, 1H, H-8), 6.45 (s, 2H, H-2′, 6′), 6.02 (s, 2H, OCH_2_O), 5.13 (dd, *J* = 9.1, 6.2 Hz, 1H, H-4), 4.38 (s, 1H, H-1), 4.29 (m, 1H, 11β-H), 4.10 (m, 1H, 11α-H), 3.81 (dd, *J* = 10.7, 2.2 Hz, 1H, H-2), 3.71 (s, 6H, 3′,5′-OCH_3_), 3.45 (m, 1H, H-3), 3.15 (s, 2H, COCH_2_), 2.58 (q, *J* = 7.0 Hz, 4H, CH_2_NCH_2_), 1.00 (t, *J* = 7.0 Hz, 6H, 2 CH_3_); ^13^C NMR (101 MHz, DMSO-*d*_6_): δ 179.08, 171.24, 148.36, 146.95, 146.87, 134.77, 131.72, 131.10, 129.48, 110.13, 105.99, 105.53, 101.51, 68.08, 56.51, 48.04, 46.35, 45.55, 43.99, 37.72, 19.03, 12.38; ESI-MS: m/z 513.5 [M+H]^+^.

##### 4β-N-[(2′′-(diethanolamine)-acetamide]-4′-demethylpodophyllotoxin (12l)

Yield 71% from **11** as a white solid; mp: 212-214 °C; ^1^H NMR (400 MHz, DMSO-*d*_6_): δ 8.29 (s, 1H, OH-4′), 8.26 (d, *J* = 8.6 Hz, 1H, CONH), 6.77 (s, 1H, H-5), 6.53 (s, 1H, H-8), 6.25 (s, 2H, H-2′, 6′), 6.01 (s, 2H, OCH_2_O), 5.20 (dd, *J* = 8.4, 4.8 Hz, 1H, H-4), 4.53 (s, 2H, 2OH), 4.48 (d, *J* = 5.2 Hz, 1H, H-1), 4.29 (m, 1H, 11β-H), 3.72 (m, 1H, 11α-H), 3.64 (s, 6H, 3′,5′-OCH_3_), 3.41 (m, 4H, 2OCH_2_), 3.36 (s, 2H, COCH_2_), 3.24 (m, 1H, H-2), 2.95 (m, 1H, H-3), 2.59 (m, 4H, 2NCH_2_); ^13^C NMR (101 MHz, DMSO-*d*_6_): δ 175.04, 171.67, 147.63, 147.59, 147.02, 135.11, 132.79, 130.69, 130.64, 109.90, 109.33, 108.89, 101.68, 68.88, 59.26, 58.81, 57.85, 56.44, 46.98, 43.44, 41.21, 37.16; ESI-MS: m/z 545.5 [M+H]^+^.

##### 4β-N-[(2′′-(diethanolamine)-acetamide]- podophyllotoxin (12m)

Yield 80% from **11** as a white solid; mp: 105-107 °C; ^1^H NMR (400 MHz, DMSO-*d*_6_): δ 8.52 (d, *J* = 9.2 Hz, 1H, CONH), 7.06 (s, 1H, H-5), 6.91 (s, 1H, H-8), 6.50 (s, 2H, H-2′, 6′), 6.03 (s, 2H, OCH_2_O), 5.11 (dd, *J* = 9.0, 6.2 Hz, 1H, H-4), 4.70 (s, 2H, 2OH), 4.30 (m, 1H, H-1), 4.07 (m, 1H, 11β-H), 3.86 (m, 1H, 11α-H), 3.73 (s, 6H, 3′,5′-OCH_3_), 3.63 (s, 3H, 4′-OCH_3_), 3.49 (m, 4H, 2OCH_2_), 3.40 (m, 1H, H-2), 3.37 (s, 2H, COCH_2_), 3.18 (m, 1H, H-3), 2.65 (m, 4H, 2NCH_2_); ^13^C NMR (101 MHz, DMSO-*d*_6_): δ 179.08, 172.13, 153.20, 147.17, 146.81, 136.60, 136.51, 131.06, 129.57, 110.23, 106.09, 105.16, 101.46, 68.16, 60.44, 59.38, 59.18, 57.72, 56.31, 46.40, 46.09, 44.01, 37.43; ESI-MS: m/z 559.6 [M+H]^+^.

##### 4β-N-[(2′′-(benzylamine)-acetamide]-4′-demethylpodophyllotoxin (12n)

Yield 85% from **11** as a white solid; mp: 141-143 °C; ^1^H NMR (400 MHz, DMSO-*d*_6_): δ 8.31 (s, 1H, OH-4′), 8.22 (d, *J* = 8.3 Hz, 1H, CONH), 7.33 (s, 2H, 3′′,5′′-ArH), 7.32 (d, *J* = 1.4 Hz, 2H, 2′′,6′′-ArH), 7.25 (m, 1H, 4′′-ArH), 6.81 (s, 1H, H-5), 6.55 (s, 1H, H-8), 6.25 (s, 2H, H-2′, 6′), 6.02 and 6.01 (2s, 2H, OCH_2_O), 5.21 (dd, *J* = 8.3, 4.7 Hz, 1H, H-4), 4.50 (d, *J* = 5.1 Hz, 1H, H-1), 4.29 (m, 1H, 11β-H), 3.76 (m, 1H, 11α-H), 3.73 (s, 2H, Ar-CH_2_), 3.71 (s, 1H, NH),3.64 (s, 6H, 3′,5′-OCH_3_), 3.22 (s, 2H, COCH_2_), 3.17 (dd, *J* = 10.6, 3.8 Hz, 1H, H-2), 2.96 (m, 1H, H-3); ^13^C NMR (101 MHz, DMSO-*d*_6_): δ 174.98, 171.11, 147.70, 147.60, 147.04, 139.93, 135.12, 132.79, 130.64, 130.59, 128.76, 128.67, 127.41, 109.91, 109.48, 108.86, 101.73, 68.83, 56.43, 52.82, 51.31, 47.15, 43.36, 41.32, 37.04; ESI-MS: m/z 547.6 [M+H]^+^.

##### 4β-N-[(2′′-(benzylamine)-acetamide]- podophyllotoxin (12o)

Yield 88% from **11** as a white solid; mp: 111-113 °C; ^1^H NMR (400 MHz, DMSO-*d*_6_): δ 8.31 (d, *J* = 8.3 Hz, 1H, CONH), 7.34 (2, 2H, 3′′,5′′-ArH), 7.32 (m, 2H, 2′′,6′′-ArH), 7.28 (m, 1H, 4′′-ArH), 6.81 (s, 1H, H-5), 6.55 (s, 1H, H-8), 6.30 (s, 2H, H-2′, 6′), 6.02 and 6.01 (2s, 2H, OCH_2_O), 5.22 (dd, *J* = 8.1, 4.6 Hz, 1H, H-4), 4.55 (d, *J* = 5.2 Hz, 1H, H-1), 4.30 (m, 1H, 11β-H), 3.79 (s, 2H, Ar-CH_2_), 3.76 (s, 1H, NH), 3.73 (m, 1H, 11α-H), 3.66 (s, 6H, 3′,5′-OCH_3_), 3.62 (s, 3H, 4′-OCH_3_), 3.28 (s, 2H, COCH_2_), 3.21 (dd, *J* = 14.5, 5.4 Hz, H-2), 2.97 (m, 1H, H-3); ^13^C NMR (101 MHz, DMSO-*d*_6_): δ 174.89, 172.51, 152.46, 147.76, 147.13, 136.78, 136.23, 132.40, 130.62, 129.00, 128.73, 127.68, 109.89, 109.56, 108.56, 101.77, 68.85, 60.35, 56.21, 52.51, 50.80, 47.16, 43.53, 41.14, 37.07; ESI-MS: m/z 561.6 [M+H]^+^.

##### 4β-N-[(2′′-(4-fluoroaniline)-acetamide]-4′-demethylpodophyllotoxin (12p)

Yield 88% from **11** as a white solid; mp: 159-162 °C; ^1^H NMR (400 MHz, DMSO-*d*_6_): δ 8.34 (d, *J* = 8.3 Hz, 1H, CO NH), 8.28 (s, 1H, OH-4′), 6.92 (t, *J* = 9.0 Hz, 2H, ArH-3′′, 5′′), 6.69 (s, 1H, H-5), 6.56 (dd, *J* = 9.0, 4.5 Hz, 2H, ArH-2′′, 6′′), 6.54 (s, 1H, H-8), 6.24 (s, 2H, H-2′, 6′), 6.01 and 5.99 (2s, 2H, OCH_2_O), 5.86 (m, 1H, H-1), 5.20 (dd, *J* = 8.3, 4.7 Hz, 1H, H-4), 4.49 (d, *J* = 5.2 Hz, 1H, 11β-H), 4.23 (m, 1H, 11α-H), 3.72 (d, *J* = 5.9 Hz, 1H, NH), 3.63 (s, 6H, 3′,5′-OCH_3_), 3.36 (s, 2H, COCH_2_), 3.17 (dd, *J* = 14.4, 5.2 Hz, 1H, H-2), 3.00–2.85 (m, 1H, H-3); ^13^C NMR (101 MHz, DMSO-*d*_6_): δ 174.95, 170.81, 156.26, 153.96, 147.67, 147.60, 147.00, 145.44, 135.12, 132.75, 130.67, 130.57, 115.74, 115.52, 113.73, 113.66, 109.86, 109.43, 108.87, 101.69, 68.74, 56.43, 47.56, 47.22, 43.38, 41.25, 36.98; ESI-MS: m/z 573.5 [M+Na]^+^.

##### 4β-N-[(2′′-(3,4-dimethoxyphenethylamine)-acetamide]-4′-demethylpodophyllotoxin (12q)

Yield 85% from **11** as a white solid; mp: 229-231 °C; ^1^H NMR (400 MHz, DMSO-*d*_6_): δ 8.60 (d, *J* = 8.2 Hz, 1H, CONH), 7.95 (s, 1H, OH-4′), 6.84 (d, *J* = 8.2 Hz, 1H, 5′′-ArH), 6.82 (d, *J* = 1.7 Hz, 1H, 2′′-ArH), 6.78 (s, 1H, H-5), 6.72 (dd, *J* = 8.2, 1.7 Hz, 1H, 6′′-ArH), 6.53 (s, 1H, H-8), 6.25 (s, 2H, H-2′, 6′), 6.01 and 5.99 (2s, 2H, OCH_2_O), 5.22 (dd, *J* = 8.2, 4.5 Hz, 1H, H-4), 4.50 (d, *J* = 5.1 Hz, 1H, H-1), 4.28 (m, 1H, 11β-H), 3.83 (m, 1H, 11α-H), 3.71 (s, 1H, NH), 3.74 (s, 6H, 3′′,4′′-OCH_3_), 3.65 (s, 6H, 3′,5′-OCH_3_), 3.53 (d, *J* = 3.9 Hz, 1H, H-3), 3.12 (dd, *J* = 14.4, 5.2 Hz, 1H, H-2), 2.96 (m, 2H, NCH_2_), 2.90 (s, 2H, COCH_2_), 2.78 (t, *J* = 7.5 Hz, 2H, Ar-CH_2_); ^13^C NMR (101 MHz, DMSO-*d*_6_) δ 174.79, 172.47, 162.70, 149.12, 147.82, 147.80, 147.59, 147.07, 135.14, 132.74, 131.36, 130.46, 130.25, 120.89, 112.83, 112.23, 108.84, 101.73, 79.75, 79.42, 79.09, 56.40, 55.91, 55.80, 49.88, 47.32, 43.36, 36.26, 31.22, 21.52; ESI-MS: m/z 643.6 [M+Na]^+^.

##### 4β-N-[2′′-(3,4-dimethoxyphenethylamine)-acetamide)]-podophyllotoxin (12r)

Yield 85% from **11** as a white solid; mp: 106-108 °C; ^1^H NMR (400 MHz, CDCl_3_): δ 7.75 (d, *J* = 8.1 Hz, 1H, CONH), 6.78 (d, 1H, H-5), 6.75 – 6.67 (m, 3H, Ar-H), 6.53 (s, 1H, H-8), 6.27 (s, 2H, H-2′, 6′), 5.97 (s, 2H, OCH_2_O), 5.21 (dd, *J* = 8.1, 4.7 Hz, 1H, H-4), 4.54 (d, *J* = 5.1 Hz, 1H, H-1), 4.40 – 4.31 (m, 1H, 11β-H), 4.13 (m, 1H, 11α-H), 4.10(s, 1H, NH), 3.92 (s, 3H, 4′-OCH_3_), 3.81 (s, 3H, Ar-OCH_3_), 3.78 (s, 3H, Ar-OCH_3_), 3.75 (s, 6H, 3′,5′-OCH_3_), 3.45 (d, 1H, H-3), 3.08 – 3.00 (m, 1H, H-2), 2.89 (s, 2H, COCH_2_), 2.84 −2.70 (m, 2H, NCH_2_), 2.65 (m, 1H, Ar-CH_2_); ^13^C NMR (101 MHz, CDCl_3_): δ 175.79, 174.44, 169.91, 152.58, 148.94, 148.22, 147.73, 147.45, 137.16, 134.93, 132.35, 130.84, 128.69, 120.70, 111.78, 111.35, 110.27, 108.84, 108.16, 101.56, 68.76, 60.77, 56.20, 55.83, 55.76, 51.11, 50.98, 47.57, 43.60, 41.54, 37.03, 34.73, 20.98; ESI-MS: m/z 635.7 [M+H]^+^.

##### 4β-N-[(2′′-(4-hydroxylphenylethylamine)-acetamide]-4′-demethylpodophyllotoxin (12s)

Yield 89% from **11** as a white solid; mp: 234-236 °C; ^1^H NMR (400 MHz, DMSO-*d*_6_): δ 9.33 (s, 1H, OH-4′), 8.73 (d, *J* = 8.2 Hz, 1H, CONH), 8.30 (s, 1H, OH-4′), 7.00 (d, *J* = 8.4 Hz, 2H, ArH-2′′, 6′′), 6.79 (s, 1H, H-5), 6.70 (d, *J* = 8.4 Hz, 2H, ArH-3′′, 5′′), 6.55 (s, 1H, H-8), 6.25 (s, 2H, H-2′, 6′), 6.02 and 6.00 (2s, 2H, OCH_2_O), 5.22 (dd, *J* = 8.2, 4.5 Hz, 1H, H-4), 4.52 (d, *J* = 5.1 Hz, 1H, H-1), 4.29 (m, 1H, 11β-H), 4.07–3.78 (m, 1H, 11α-H), 3.64 (s, 6H, 3′,5′-OCH_3_), 3.59 (s, 2H, COCH_2_**)**, 3.19 (dd, *J* = 14.4, 5.2 Hz, 1H, H-2), 3.00(m, 1H, H-3), 2.94 (t, 2H, NCH_2_), 2.75 (t, 2H, CH_2_-Ar), 1.92(s, 1H, NH); ^13^C NMR (101 MHz, DMSO-*d*_6_): δ 174.89, 156.43, 147.77, 147.61, 147.02, 135.13, 132.80, 130.54, 130.29, 129.95, 128.57, 115.75, 109.97, 109.47, 108.86, 101.76, 68.71, 56.43, 49.73, 49.55, 47.37, 43.31, 41.38, 36.94, 32.64; ESI-MS: m/z 599.6 [M+Na]^+^.

##### 4β-N-[2′′-(4-hydroxylphenylethylamine)-acetamide)]-podophyllotoxin (12t)

Yield 81% from **11** as a white solid; mp: 116-119 °C; ^1^H NMR (400 MHz, CDCl_3_): δ 8.01 (s, 1H, Ar-OH), 7.80 (d, *J* = 9.0 Hz, 1H, CONH), 6.99 (d, *J* = 8.3 Hz, 2H, Ar-H), 6.71 (d, *J* = 8.3 Hz, 2H, Ar-H), 6.67 (s, 1H, H-5), 6.63 (s, 1H, H-8), 6.36 (s, 2H, H-2′, 6′), 5.97 and 5.96 (2s, 2H, OCH_2_O), 5.30 (dd, *J* = 9.1, 5.1 Hz, 1H, H-4), 4.43 (d, 1H, H-1), 4.20 (m, 1H, 11β-H), 4.14 (m, 1H, 11α-H), 3.82 (s, 3H, 4′-OCH_3_), 3.79 (s, 6H, 3′,5′-OCH_3_), 3.44 (dd, *J* = 10.3, 2.8 Hz, 1H, H-2), 3.36 (d, 2H, COCH_2_), 3.24 (m, 1H, H-3), 2.93 (s, 1H, NH), 2.86 (d, *J* = 4.0 Hz, 2H, NCH_2_), 2.70 (t, *J* = 7.1 Hz, 2H, CH_2_-Ar); ^13^C NMR (101 MHz, CDCl_3_): δ 179.01, 171.69, 155.06, 153.47, 147.62, 147.43, 137.37, 136.84, 130.23, 130.16, 129.77, 128.22, 116.26, 116.18, 115.80, 115.60, 115.57, 110.02, 106.08, 104.79, 101.41, 68.51, 60.87, 56.22, 51.56, 51.35, 47.30, 45.41, 45.31, 37.88, 34.97, 29.71, 14.20; ESI-MS: m/z 591.6 [M+H]^+^.

### Biological Evaluation

#### Cell Culture

The four human cancer cell lines and a normal human epidermal cell line of the screening panel, including H460 (human non-small cell lung carcinoma), HeLA (human cervical carcinoma), EC9706 (human esophageal squamous cell carcinoma), T24 (human bladder carcinoma), and HaCaT (human immortalized epidermal cells), were purchased from American Type Culture Collection (ATCC, Manassas, VA, U.S.A.). H460, EC9706, and T24 were maintained in RPMI 1640 medium containing 10% Fetal Bovine Serum, 100 units/ml penicillin, 100 μg/ml streptomycin under humidified incubator with 5% CO_2_ atmosphere at 37°C. HeLA and HaCaT were maintained in Dulbecco′s Minimum Essential Medium (DMEM) supplemented with 10% Fetal Bovine Serum 100 units/ml penicillin, 100 μg/ml streptomycin in a humidified incubator and 5% CO_2_ atmosphere at 37°C. Logarithmically growing cells were used for the following experiments.

#### Antiproliferative Assay

The cytotoxicity of the synthesized compounds **12a–t** against a panel of human cell lines was determined by the 3-(4,5-dimethylthiazol-2-yl)-2,5-diphenyltetrazoliun bromide (MTT) growth inhibition assay. The five human cell lines were, respectively, plated in 96-well-culture plates at the density of 1 × 10^5^ cells per well and incubated for 24 h. Cells were exposed to different concentrations of synthetic podophyllotoxin derivatives for 48 h. MTT was added with a dose of 5 mg/mL in phosphate-buffered saline. After incubation for 4 h at 37°C, the purple formazan crystals were dissolved with 100 mL dimethyl sulfoxide and the absorbance was measured at 570 nm in an ELISA reader. Antiproliferative activity was expressed using the IC_50_ value defined as the concentration of synthetic podophyllotoxin derivatives inhibiting cell proliferation by 50%. The cell viability ratio was calculated by the following formula: cell viability ratio (%) = OD treated /OD control × 100% (Ma et al., 2014).

#### Hoechst 33258 Staining

T24 and HeLA cells were seeded at a density of 3 × 10^4^ cells per well and cultured in 6 well-plates on a cover slip for 24 h at 37°C. Compound **12g** treated T24 and HeLA cancer cells for 24 h at 37°C with concentrations of 0.5/1.0/2.0 and 0.75/1.5/3.0 μM, respectively. Afterward, the treated cells were fixed with 4% paraformaldehyde (PFA) for 30 min and stained with 5 μg/mL Hoechst 33258 (bis-benzimide; KeyGEN Bio TECH, China) for 30 min. Nuclei were stained with Hoechst 33258 to examine chromatin condensation or nuclear fragmentation, morphological characteristics of apoptosis. After the cells were washed twice with PBS, the cover slip was inverted and placed on a glass slide and mounted. Apoptotic cells with fluorescence of the soluble DNA fragments were detected directly and photographed under a phase contrast microscope (OLYMPUS IX51, Japan) in a Varian Fluorometer at an excitation wavelength of 365 nm and emission wavelength of 460 nm (Shareef et al., 2015).

#### Cell Cycle Distribution Analysis

To understand the cell cycle effect of the synthesized analogs, cell cycle distribution analysis was performed by FACS (Becton Dickinson, San Jose, CA, USA). T24 and HeLA cells were treated with compound **12g** for 24 h at 37°C with concentrations of 0.5/1.0/2.0 and 0.75/1.5/3.0 μM, respectively. After treatment, the cells were washed once with PBS and fixed with 70% ice-cold ethanol at 20°C for overnight. Ethanol was removed by Centrifugation. The cells were stained with a solution containing 0.1% Triton-X 100 (Sigma), 0.2 mg/mL RNase (Sigma), and 20 mg/mL propidium iodide (PI, Sigma) in the dark for 30 min at room temperature. Then, cell cycle distribution was analyzed by using a FACS can flow cytometer (Chen et al., 2013).

### Molecular Docking Study

Docking study simulations were performed using AutoDock 4.0 to investigate the potential binding mode of the synthesized compound **12g** in the active site of human DNA topoisomerase IIα (PDB: 1ZXM, Available from: https://www.rcsb.org/structure/1ZXM) and to predict its mechanism of action as an anti-cancer agent. Autogrid was employed using a grid box volume of 50 × 50 × 50 Å centered on the active site of human DNA topoisomerase IIα. The 3D structures of the synthesized compounds were employed to achieve the docking study (Chen et al., 2013; Shareef et al., 2015). The docking protocol was then applied and 100 poses per compound were generated, and the best docked structure was chosen to fulfill the docking procedure. The docking protocol mainly consisted of four steps. (1) Ligand preparation: Chemidraw 11.0 was employed to process the structure of small molecule **12g**; after energy minimization optimization, the small molecule was saved in the mo12 format, which was then converted into a pdbqt file by Autodock 4.0. (2) Receptor preparation: after removal of water molecules, its natural ligand and excess protein chains in the structure of 1ZXM downloaded from the pdb database, the protein 1ZXM was processed with Autodock 4.0. via hydrogenation, calculation of charge, and combination of non-polar hydrogen, which was saved as pdbqt file. (3) Autogrid processing: the pdbqt file of protein 1ZXM was processed by autogrid to construct a 50 × 50 × 50 box centered on the active site of the protein, which generated a glg file. (4) Autodock operation: using the default software parameters, the small molecule was autodocked with the protein in flexible docking, and the operation was processed 100 times to generate a dlg file. The final figures of the molecular modeling were visualized using PYMOL.

## Author Contributions

JW was responsible for the experimental implementation and paper writing. JC, PJ, LM, LC, WM, and TZ provided literature retrieval and guidance for methods. GY was responsible for the coordination of this study. Y-XW was responsible for the paper editing.

### Conflict of Interest Statement

The authors declare that the research was conducted in the absence of any commercial or financial relationships that could be construed as a potential conflict of interest.
