# Supplementary material for: Synthesis and Biological Evaluation of 4β-N-Acetylamino Substituted Podophyllotoxin Derivatives as Novel Anticancer Agents
Source: Front Chem. 2019 Apr 24;7:253. doi: 10.3389/fchem.2019.00253 (PMC6491884; doi:10.3389/fchem.2019.00253)
Supplement: Supplementary file 1 [file Table_1.DOCX]

Electronic Supplementary Material (ESI).

Supporting Information

**Synthesis and biological evaluation of 4β-N-acetylamino substituted podophyllotoxin derivatives as novel anticancer agents**

Jinbao Wei ^a, b^, Jinghong Chen ^c^, Peijun Ju ^c^, Le Ma ^a^, Li Chen ^b^, Weidong Ma ^b^, Tao Zheng ^b^, Guangyi Yang ^d,^ * and Yongxiang Wang ^a,^ *

*^a^ School of Pharmacy, Shanghai Jiao Tong University, Shanghai 200240, P.R. China*

*^b^ Institute of Wudang Herbal Medicine Research, Department of Pharmacy, Taihe hospital, Hubei University of Medicine, Shiyan, Hubei 442000, P.R. China*

*^c^ Shanghai Mental Health Center, School of Medicine, Shanghai Jiao Tong University. Shanghai 200030, P.R. China*

*^d^ Baoan Hospital of Traditional Chinese Medicine, Shenzhen, Guangdong 518000, P.R. China*

* *Corresponding authors. E-mail: yxwang@sjtu.edu.cn (Y. Wang);* *[ygy996@163.com](mailto:ygy996@163.com) (G. Yang.)*

**CONTENT：**

1 Experimental Section……………………………..………………………………… S2

2 ^1^H, ^13^C NMR Spectra……………………..…………………………………… S3-S22

**1. Experimental Section**

**1.1 General method**

All solvents, reagents, and chemicals for the synthesis of the compounds were of analytical grade, purchased from commercial sources and used without further purification, unless otherwise specified. Melting points were taken on a Kofler melting point apparatus and are uncorrected. ^1^H NMR and ^13^C NMR spectra were measured on a Bruker Ascend^TM^-400 spectrometer (Bruker Company, USA) with tetramethylsilane (TMS) as an internal standard. All chemical shift values are expressed in d parts per million. Mass spectra were recorded on a Waters-XEVO UPLC/MS/MS spectrometer with ESI source as ionization. Podophyllotoxin (PPT, **1**) and 4'-demethylpodophyllotoxin (DPPT, **2**) were isolated from the Chinese medicinal herb *Dysosma versipellis*, and served as the starting materials for the preparation of all new derivatives.

**1.2 General synthetic procedure**

**General synthetic procedure for the key intermediates 4β-chloroamido-podophyllotoxin (11) and 4β-chloroamido-4'-demethylpodophyllotoxin (11a)** To a stirred mixture of PPT or DPPT (4 mmol) and ClCH2CN (10 mL), a homogeneous mixture of MsOH/Al_2_O_3_ (60 mass %, 1 g) was added, and the mixture was irradiated by an ultrasonic generator in a water bath at 60°C for 30 min then evaporated under reduced pressure. The residue was purified by chromatography on silica gel using EtOAc–petroleum ether to give the key intermediate **11** or **11a.**

**General synthetic procedure for compounds 12a-t** The key intermediate **11** or **11a** (1.0 mmol) was add to a solution of various substituted amines (1.2 mmol), potassium iodate (0.1 mmol) and potassium carbonate (2.4 mmol) in dry acetonitrile (10 mL). The reaction mixture was stirred for 2.5 h at 65°C then evaporated under reduced pressure. The residue was purified by chromatography on silica gel using EtOAc–petroleum ether to give compounds **12a–t**.

**1.3 Structure identification**

Here, we took the active compound **12g** as an example. In our data of NMR, the coupling constants of H-4 with H-3 and NH was 8.4 and 4.8 Hz, which were consistent with the analogues in previous reported literatures (Z.-J. Zhang et al. / Bioorg. Med. Chem. 22 (2014) 204–210; W.-Q. Li et al. / Bioorg. Med. Chem. 21 (2013) 2363–2369). In the more stable conformation of demethylepipodophylotoxins H4-alpha is equatorial, H3-alpha is axial, H2-beta is axial and H1-beta is equatorial. Axial-axial vicinal coupling constants are usually bigger than axial-equatorial coupling constants. Furthermore, in cyclohexane stereoisomer, the coupling constant of vicinal axial-H with equatorial-H is less than 12 Hz, whereas the coupling constant of axial-axial is over 12 Hz. Hence, the synthesized compounds were cis-configuration between C3 and C4.The coupling constants of H-3 with H-2 is 14.5 Hz, suggesting the configuration of C2 and C3 is trans-form. The coupling constant of H-2 with H-1 is 5.2 Hz, indicating the configuration of C2 and C3 is cis-form. In combination with the NMR data of etoposide, we could confirm that the synthesized compounds C4-β derivatives and that the spatial configuration was as follow: the configuration of H4 and H3 was cis, the configuration of H3 and H2 was trans, the configuration of H2 and H1 was cis.

**14b** 4β-N-(Ethylsulfonylurea)-4-deoxyepipodophyllotoxin (Z.-J. Zhang et al. / Bioorg. Med. Chem. 22 (2014) :204–210) ^1^H NMR (400 MHz, DMSO-d6): δ 6.87 (s, 1H, 5-H), 6.54 (s, 1H, 8-H), 6.26 (s, 2H, 20,60-H), 6.00 and 5.99 (ABq, 2H, –OCH2O–), 5.01 (dd, 1H, 4-H, J = 8, 4.8 Hz), 4.54 (d, 1H, 1-H, J = 5.2 Hz), 4.34 (t, 1H, 11b-H, J = 8 Hz), 3.79 (t, 1H, 11a-H, J = 10.4 Hz), 3.63 (s, 6H, 30,50-OCH3), 3.60 (s, 3H, 40-OCH3), 3.20 (dd, 1H, 2-H, J = 14.4, 5.2 Hz), 2.99–2.89 (m, 1H, 3-H), 1.23 (t, 3H, 200-H, J = 7.6 Hz).

**12g** (4β-N-[(2''-( dipropylamine)-acetamide]-4'-demethylpodophyllotoxin)：^1^H NMR (400 MHz, DMSO-d6): δ 8.29 (s, 1H, CONH), 8.12 (s, 1H, OH-4'), 6.77 (s, 1H, H-5), 6.55 (s, 1H, H-8), 6.25 (s, 2H, H-2', 6'), 6.01 and 5.99 (2s, 2H, OCH2O), 5.19 (dd, J = 8.4, 4.8 Hz, 1H, H-4), 4.51 (d, J = 5.2 Hz, 1H, H-1), 4.31 (t, J = 8.0 Hz, 1H, 11β-H), 3.75 (m, 1H, 11α-H), 3.64 (s, 6H, 3',5'-OCH3), 3.37 (s, 2H, COCH2), 3.20 (dd, J = 14.5, 5.2 Hz, 1H, H-2), 3.03–2.92 (m, 1H, H-3), 2.53 (s, 4H, 2NCH2), 1.50–1.35 (m, 4H, 2CH2), 0.80 (t, J = 7.3 Hz, 6H).

**Etoposide** (SDBS, Web : <http://sdbs.riodb.aist.go.jp>) ^1^H NMR (400 MHz, DMSO-d6): δ 8.22 (s, 1H, A: 4'OH), 7.004 (s, 1H, B: H-5), 6.524 (s, 1H, C: H-8), 6.183 (s, 2H, D: H-2', 6'), 6.023-6.021 (2s, 2H, E/F: OCH2O), 5.21-5.21 (dd,2H, G/J: Glu-OH), 4.931 (d, 1H, K: H-4), 4.722 (q, 1H, L: Glu-H), 4.573 (d, 1H, M: Glu-H), 4.490 (d, 1H, N: H-1), 4.27 (d, 1H, P: 11β-H), 4.25(d, 1H, Q: 11α-H), 4.078(m, 1H, R: Glu-H), 3.612(s, 6H, S: 3',5'-OCH3), 3.50 (t, 1H, T: Glu-H), 3.34(m, 1H, U: Glu-H), 3.27(m, 1H, V: H-2), 3.25(m, 1H, W: Glu-H), 3.167(m, 1H, X: Glu-H), 3.068(m, 1H, Y: Glu-H), 2.883(m, 1H, Z: H-3), 1.239(d, 3H, AA: Glu-CH3).

**2. ^1^H, ^13^C NMR Spectra**

**12a:**

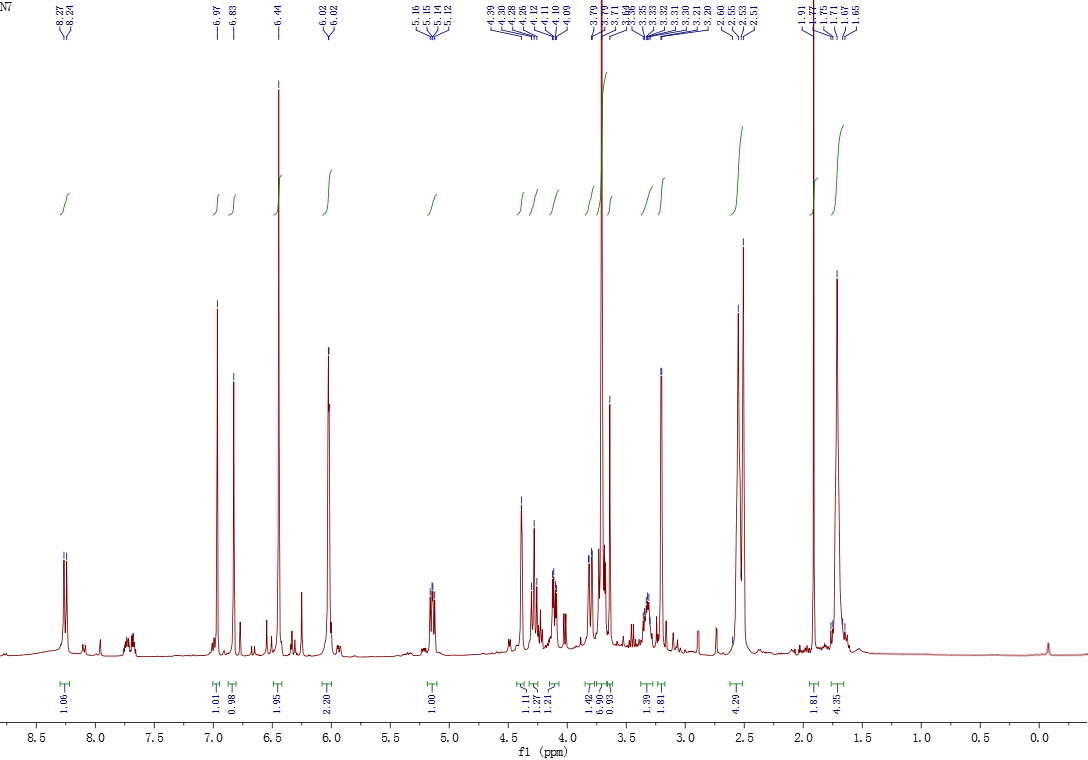


**^1^H NMR**

**
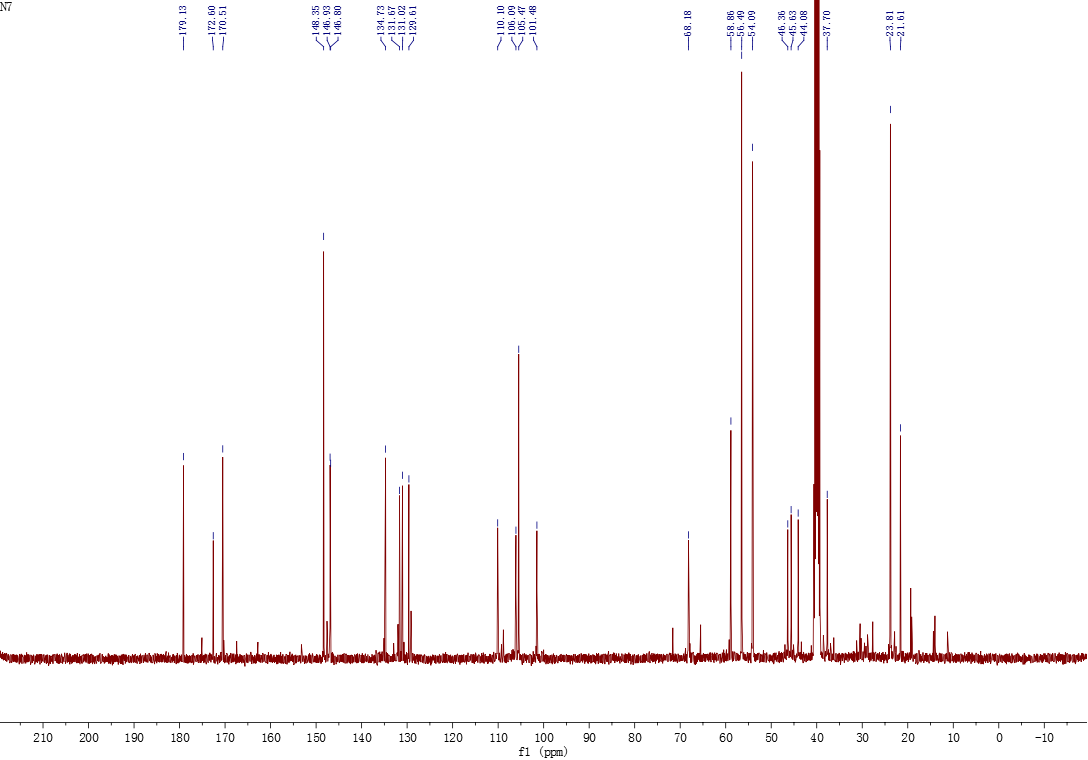
**

**^13^C NMR**

**12b:**

**
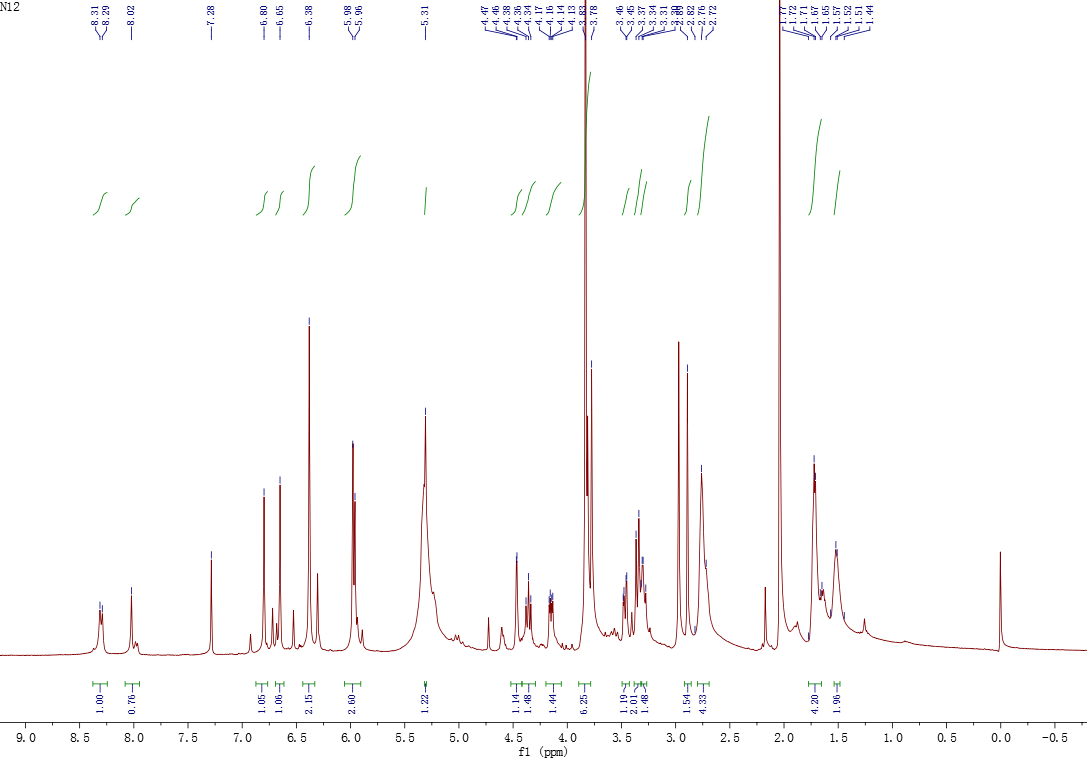
**

**^1^H NMR**

**
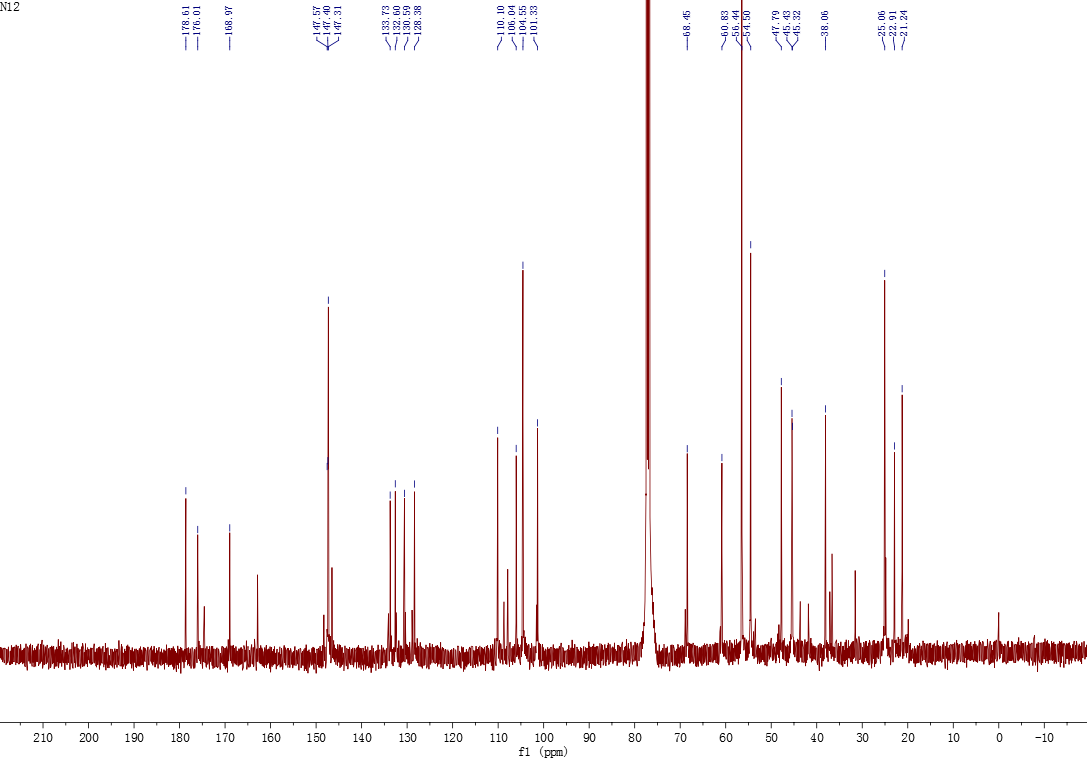
**

**^13^C NMR**

**12c:**

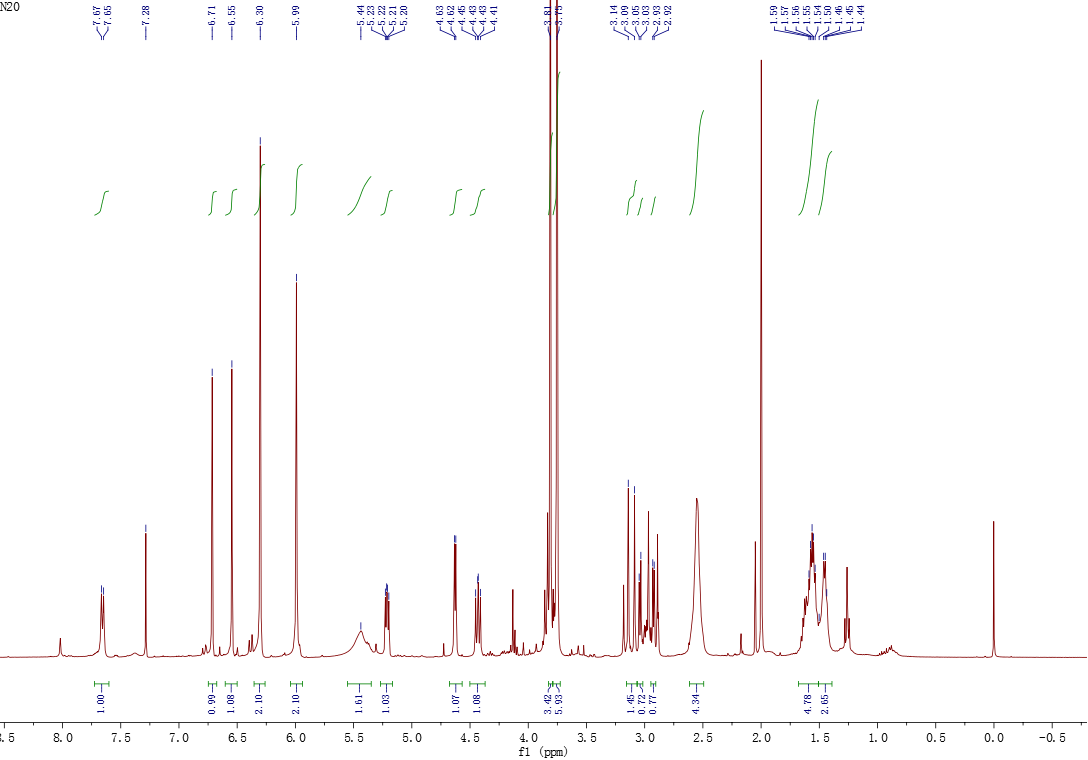


**^1^H NMR**


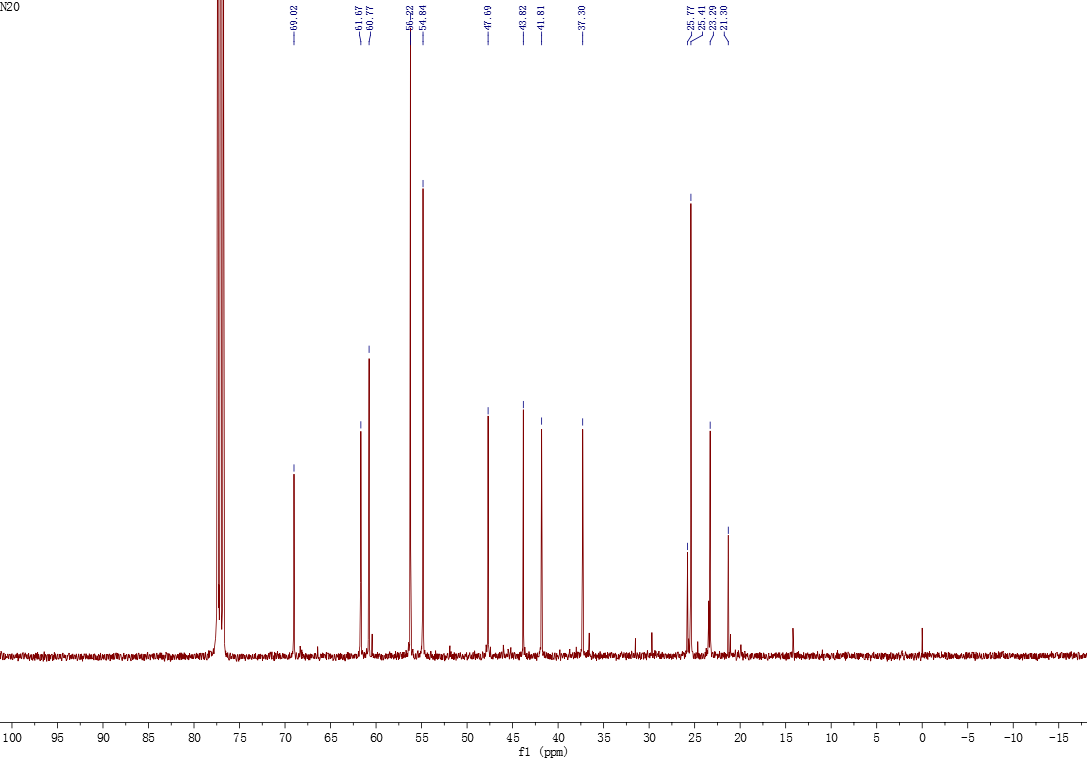


**^13^C NMR**

**12d:**

**
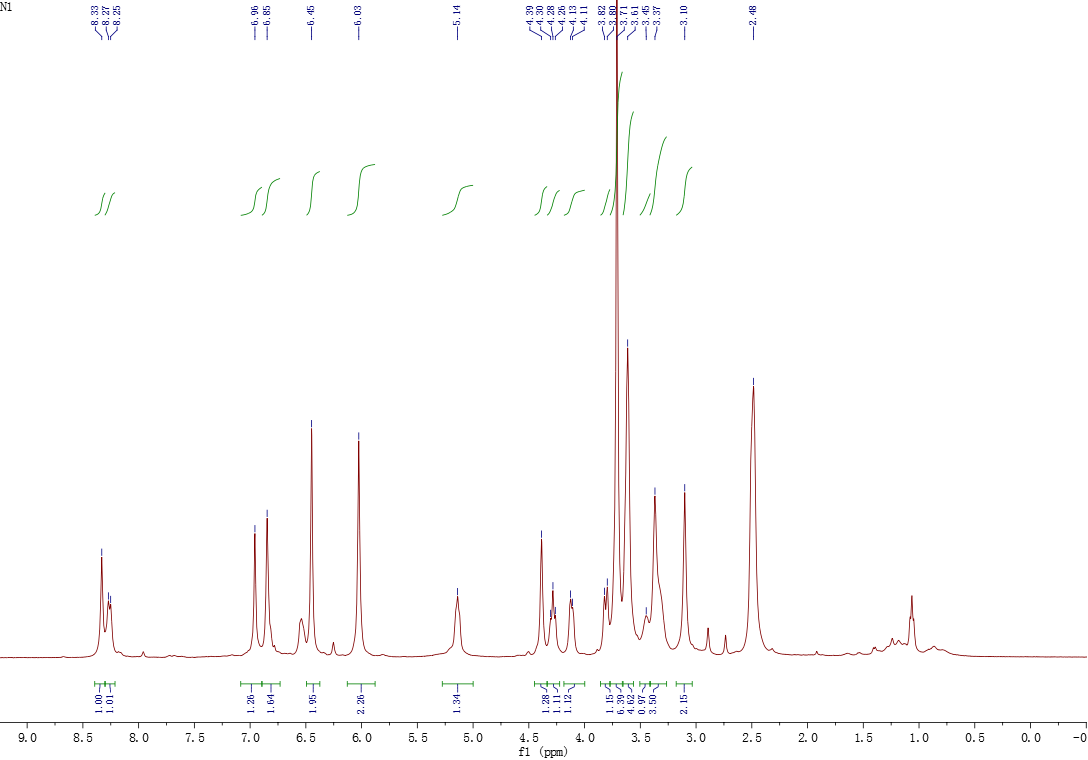
**

**^1^H NMR**

**
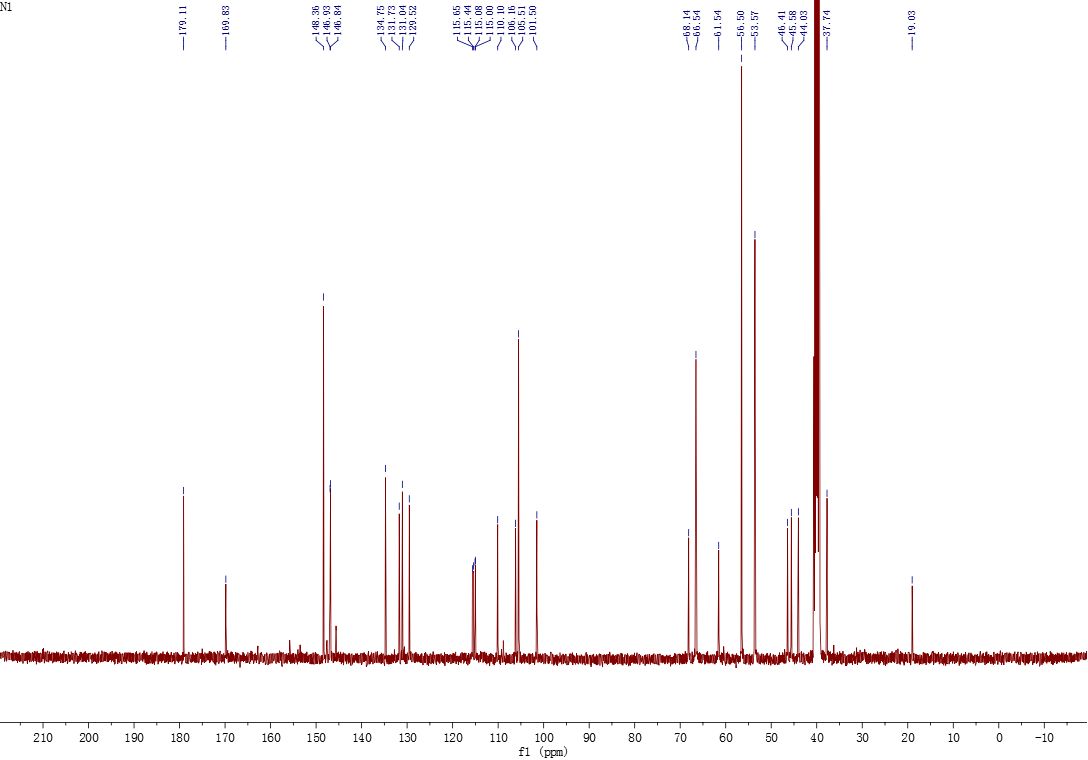
**

**^13^C NMR**

**12e:**


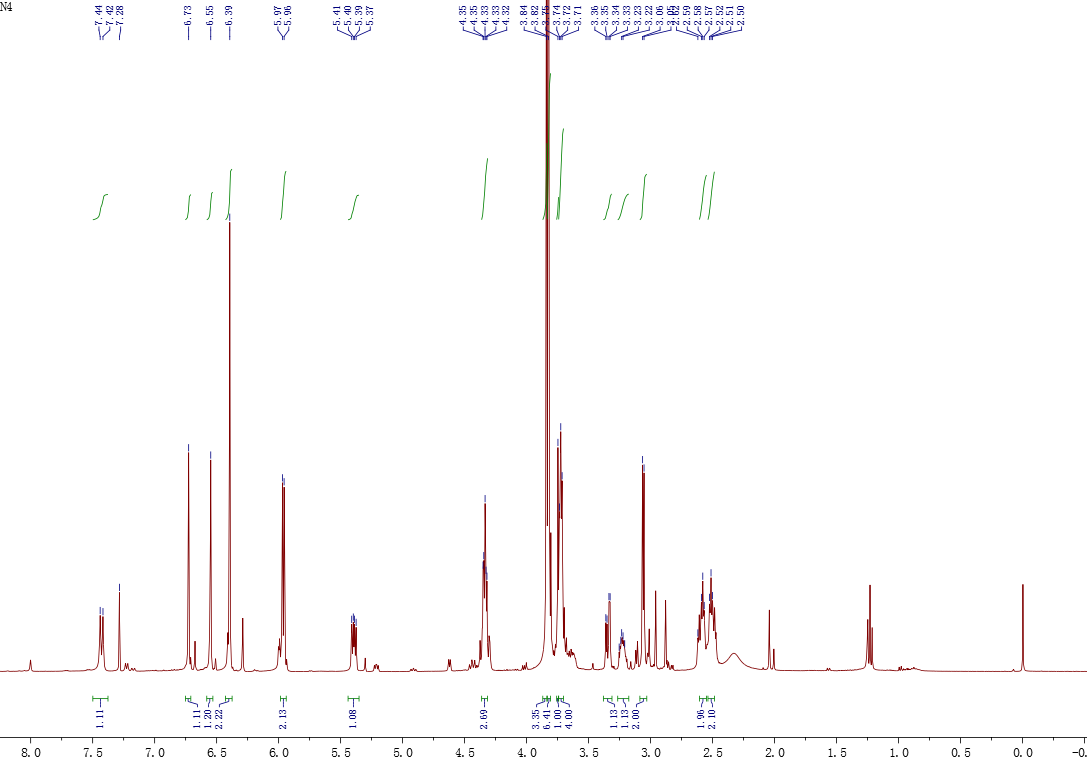


**^1^H NMR**

**
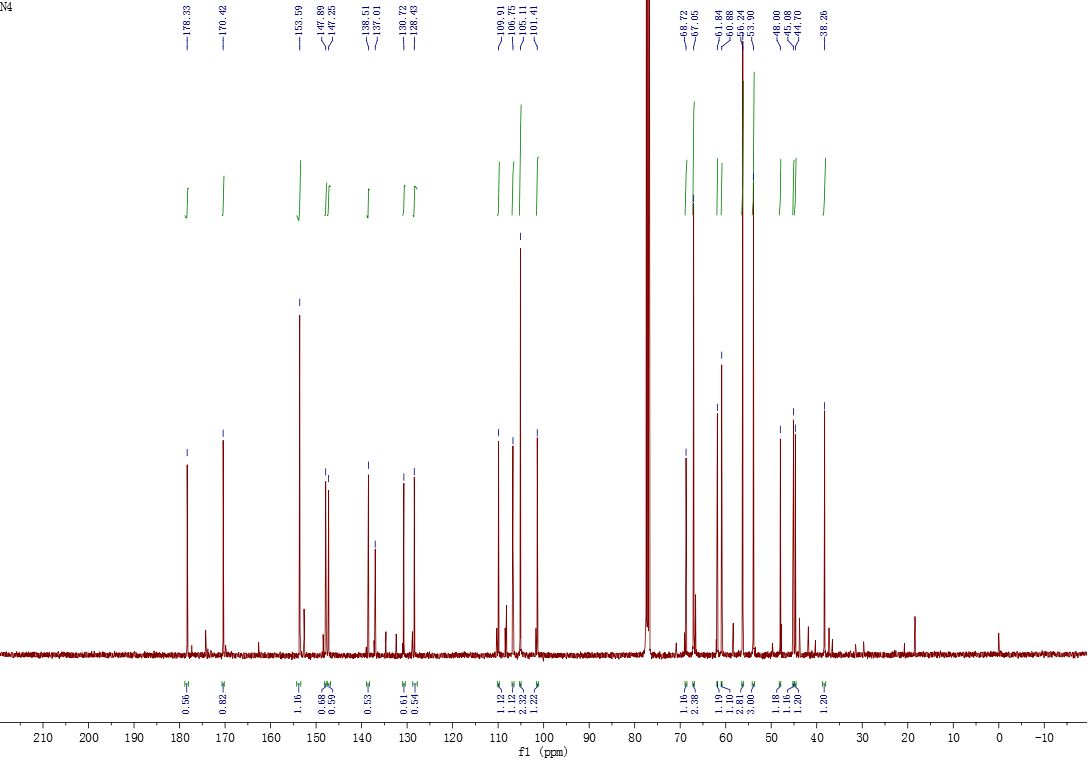
**

**^13^C NMR**

**12f:**

**
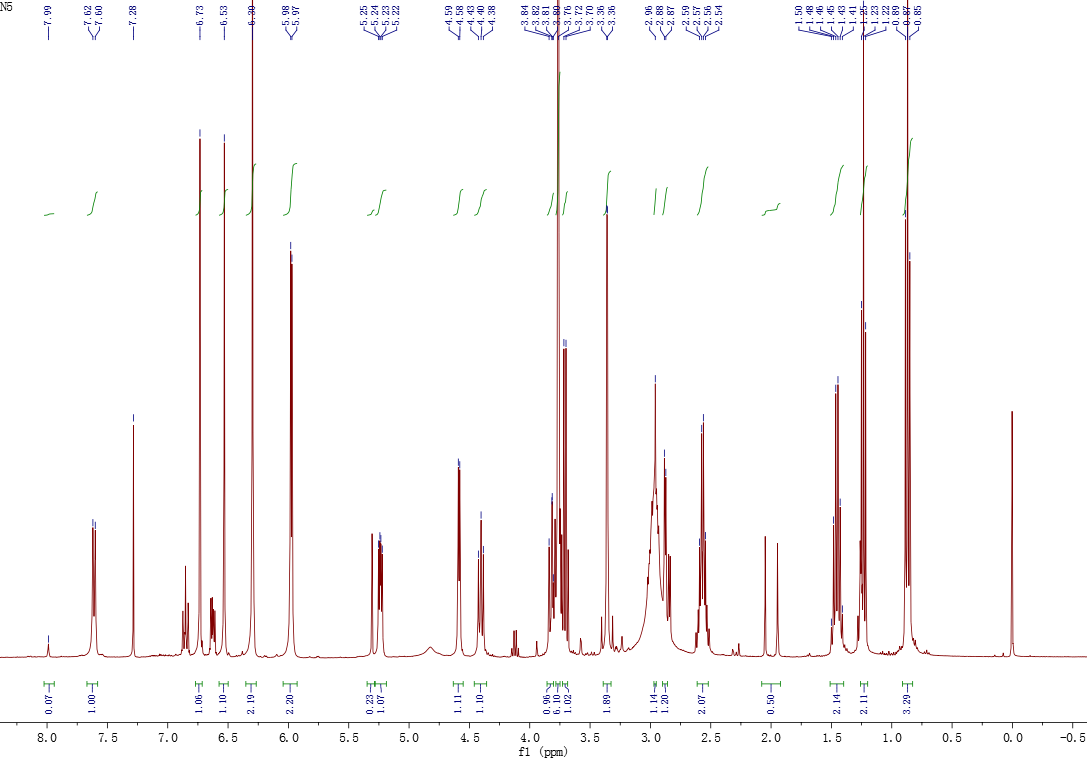
**

**^1^H NMR**

**
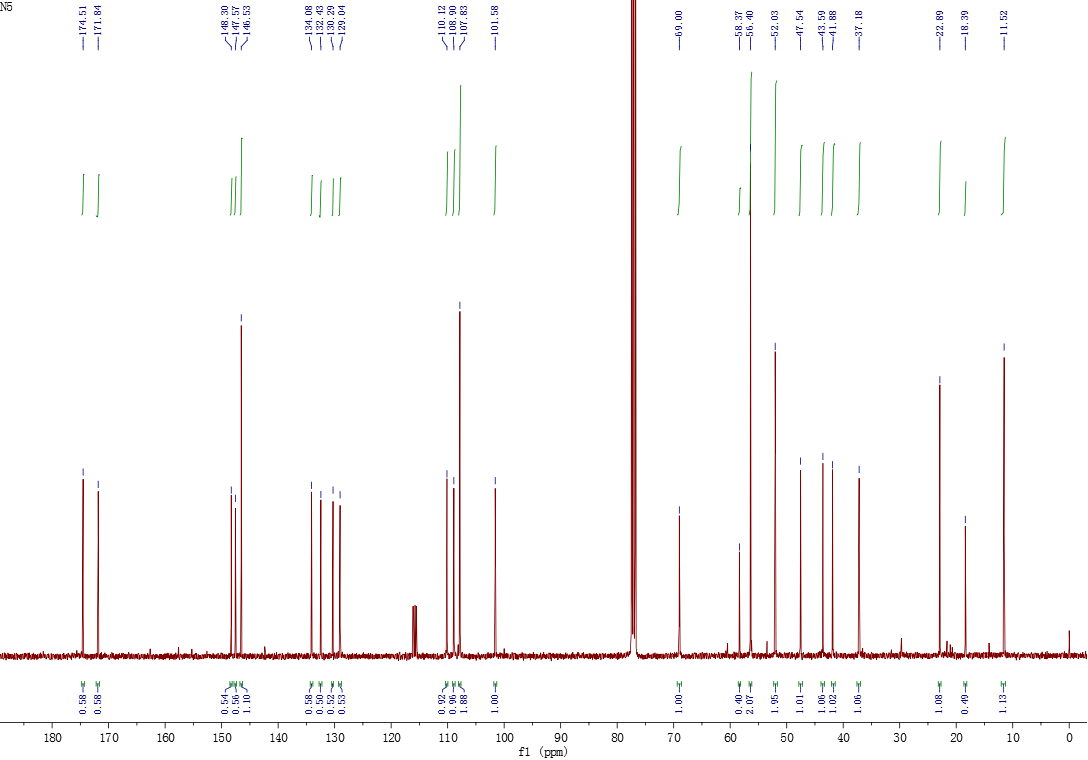
**

**^13^C NMR**

**12g：**

**
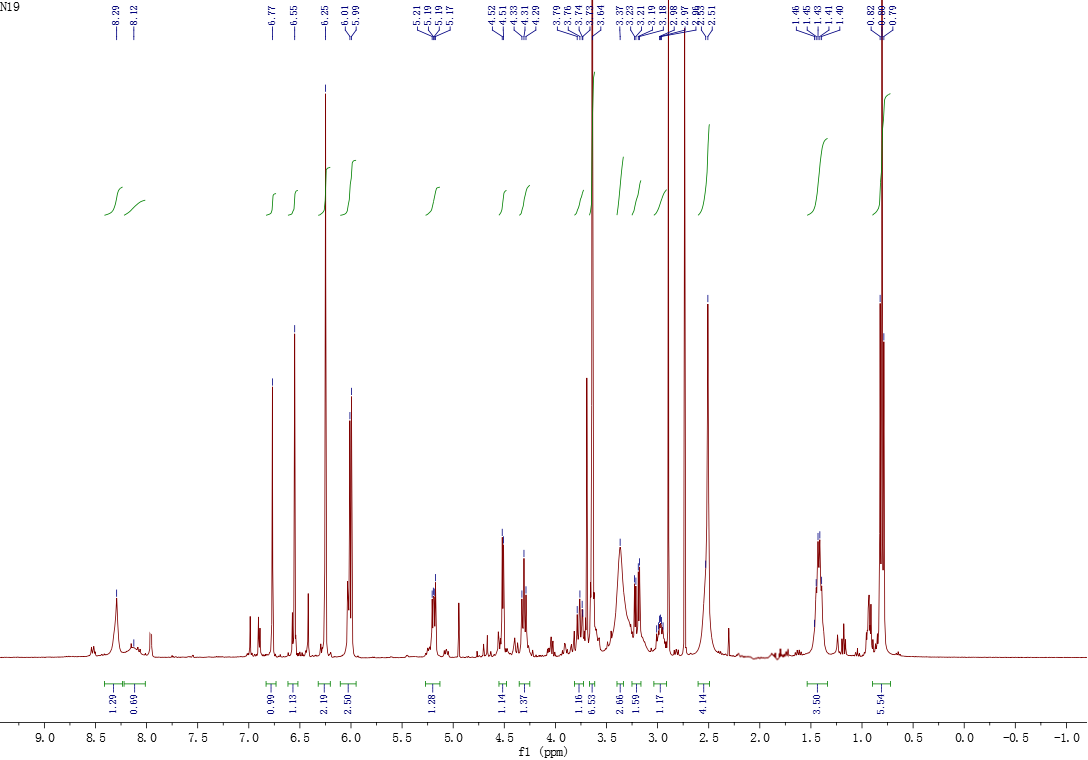
**

**^1^H NMR**

**
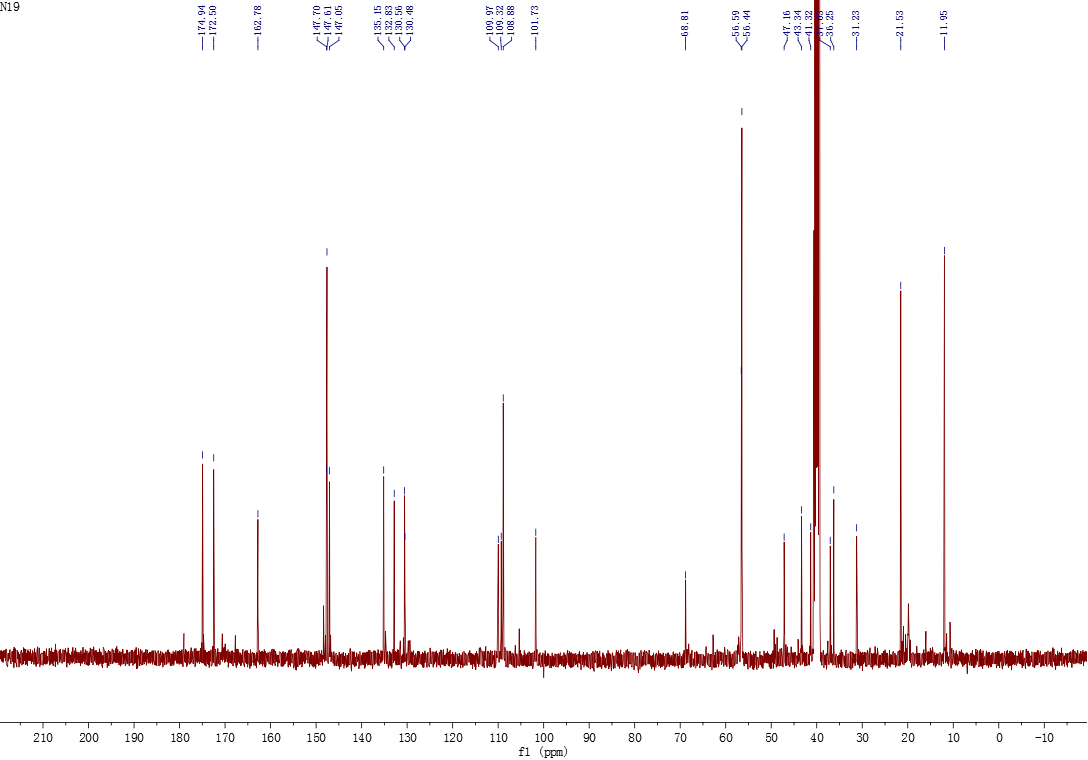
**

**^13^C NMR**

**12h：**

**
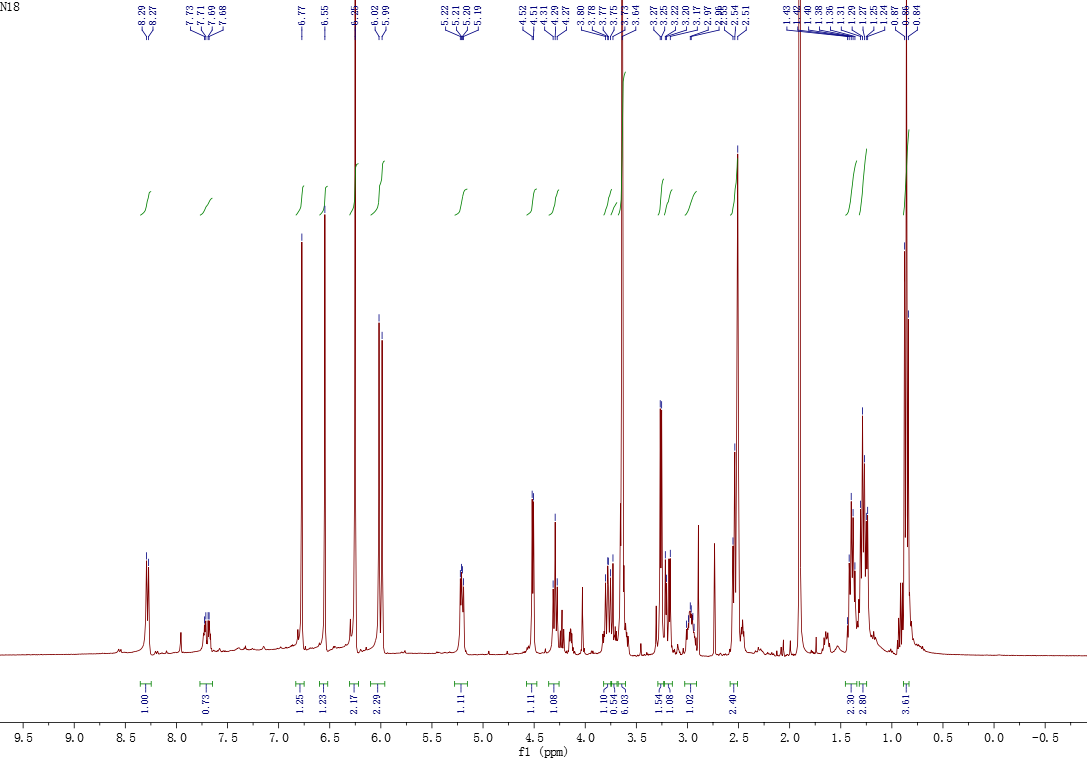
**

**^1^H NMR**

**
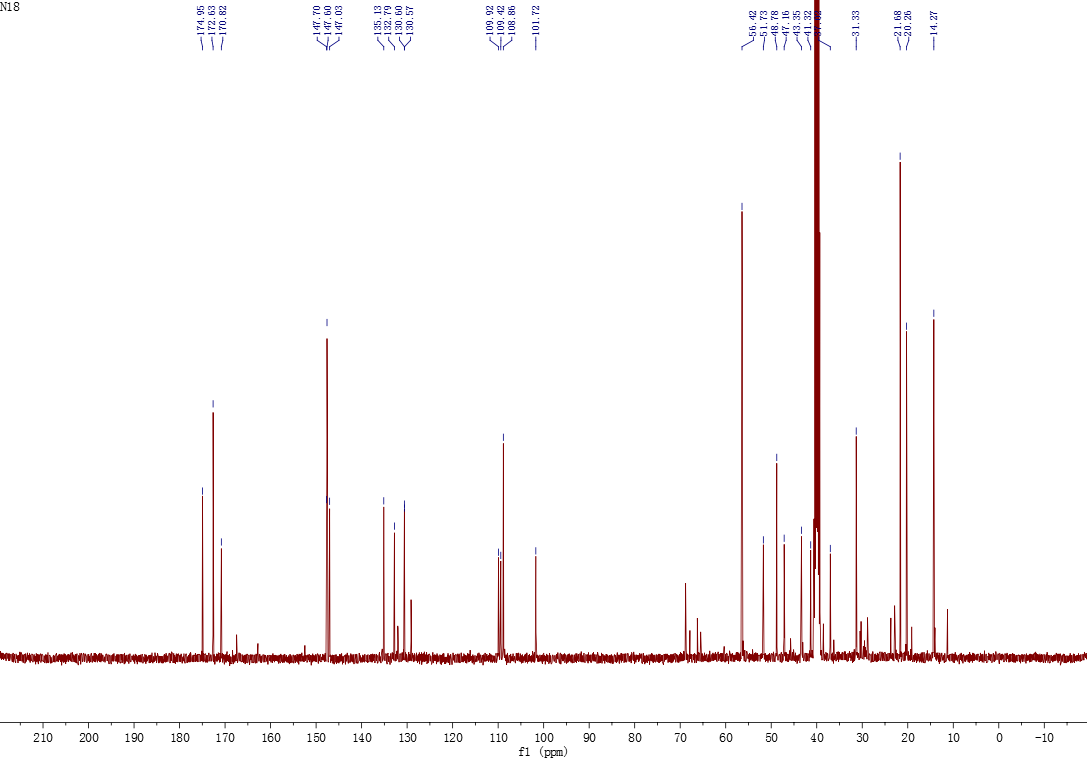
**

**^13^C NMR**

**12i：**

**
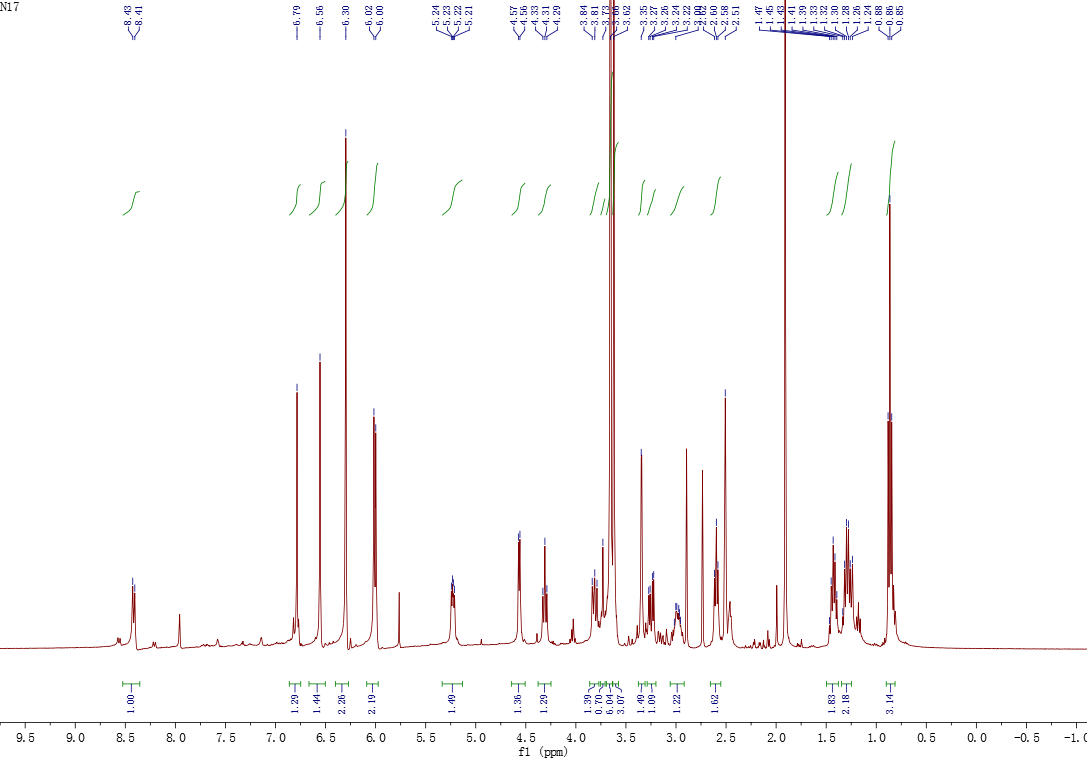
**

**^1^H NMR**

**
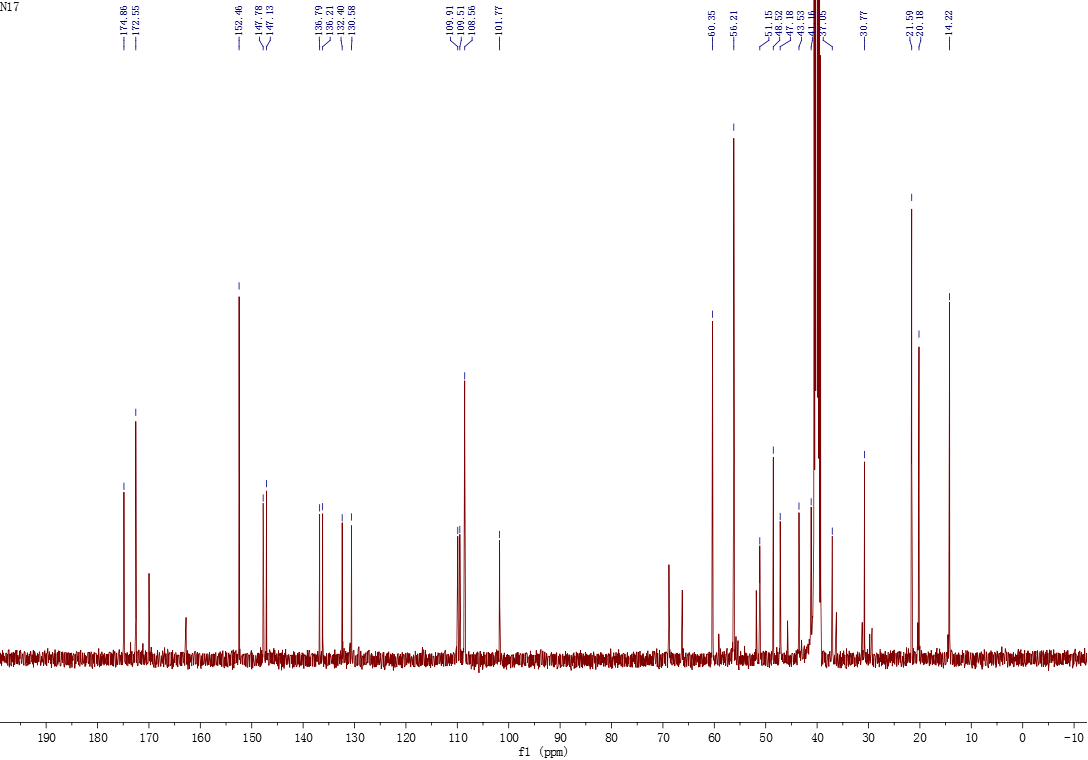
**

**^13^C NMR**

**12j：**

**
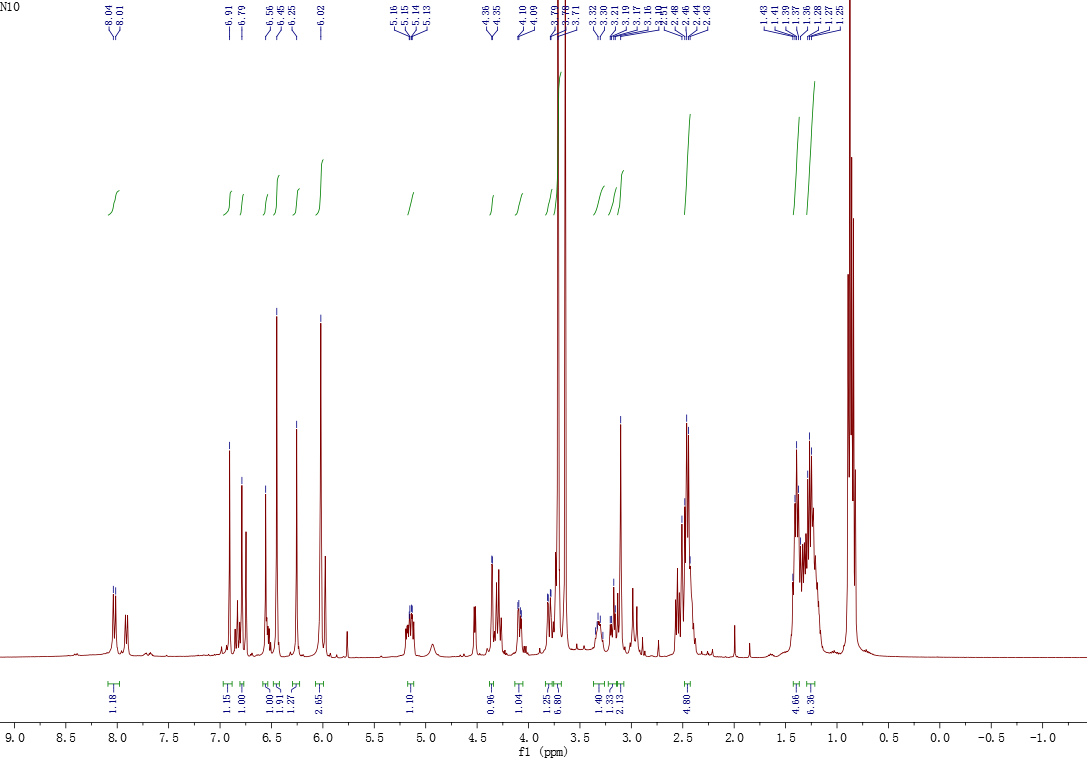
**

**^1^H NMR**

**
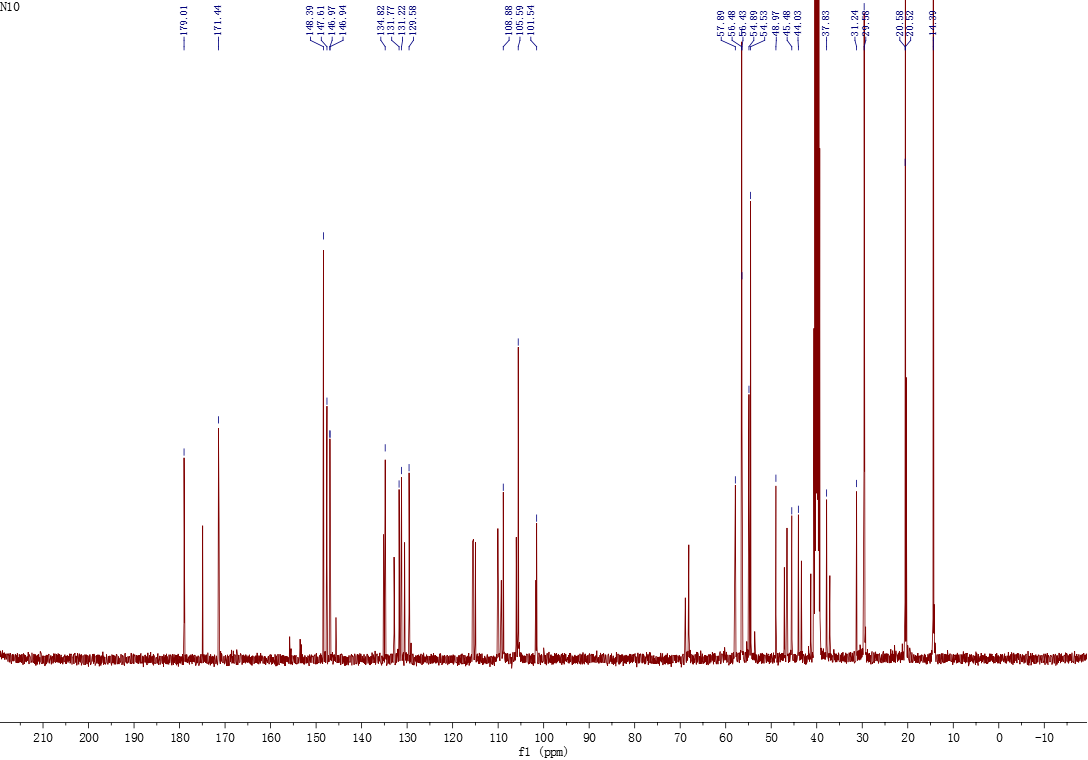
**

**^13^C NMR**

**12k：**

**
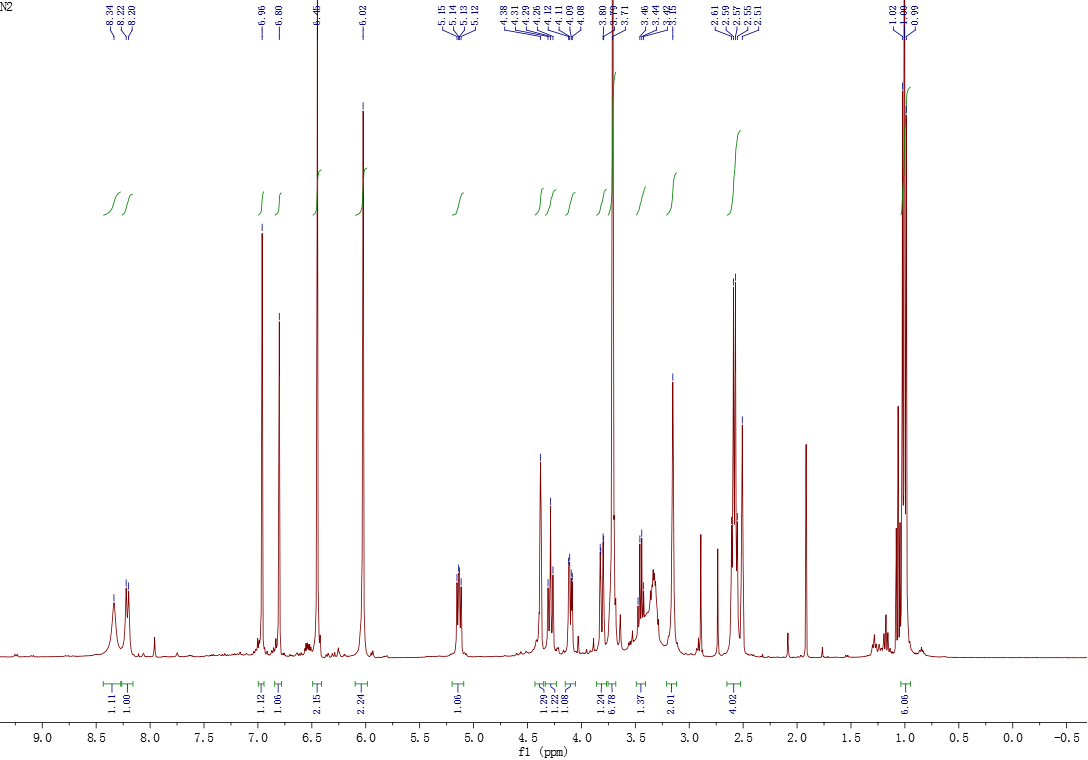
**

**^1^H NMR**

**
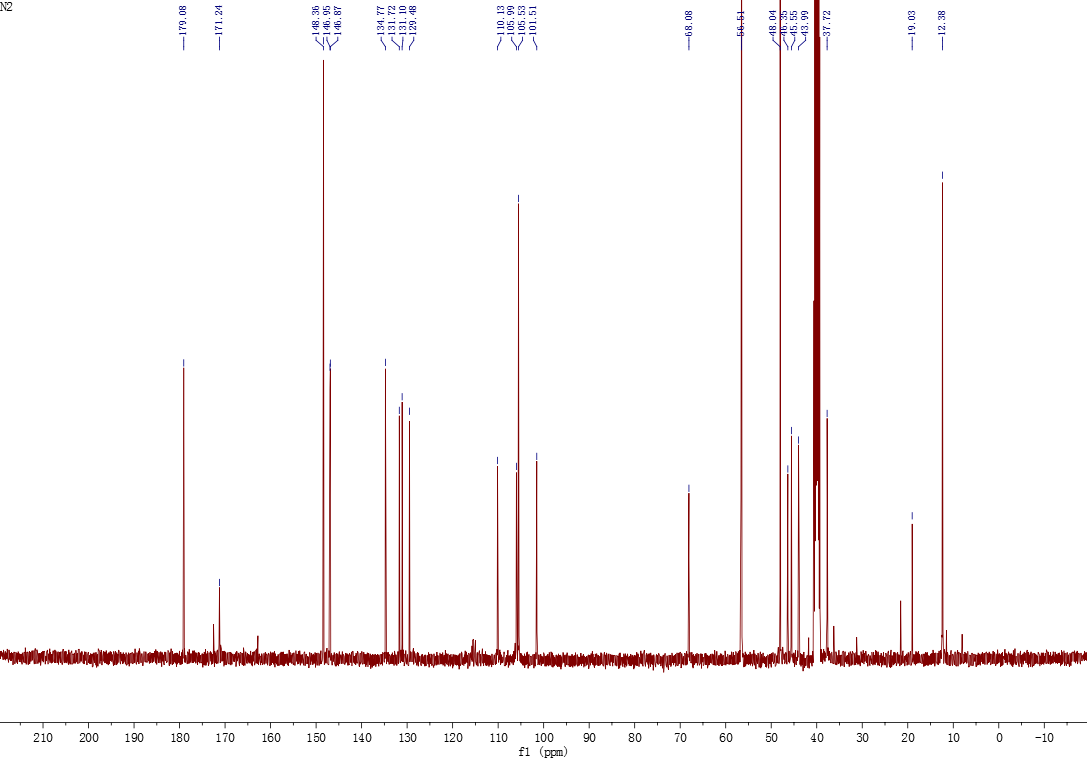
**

**^13^C NMR**

**12l：**

**
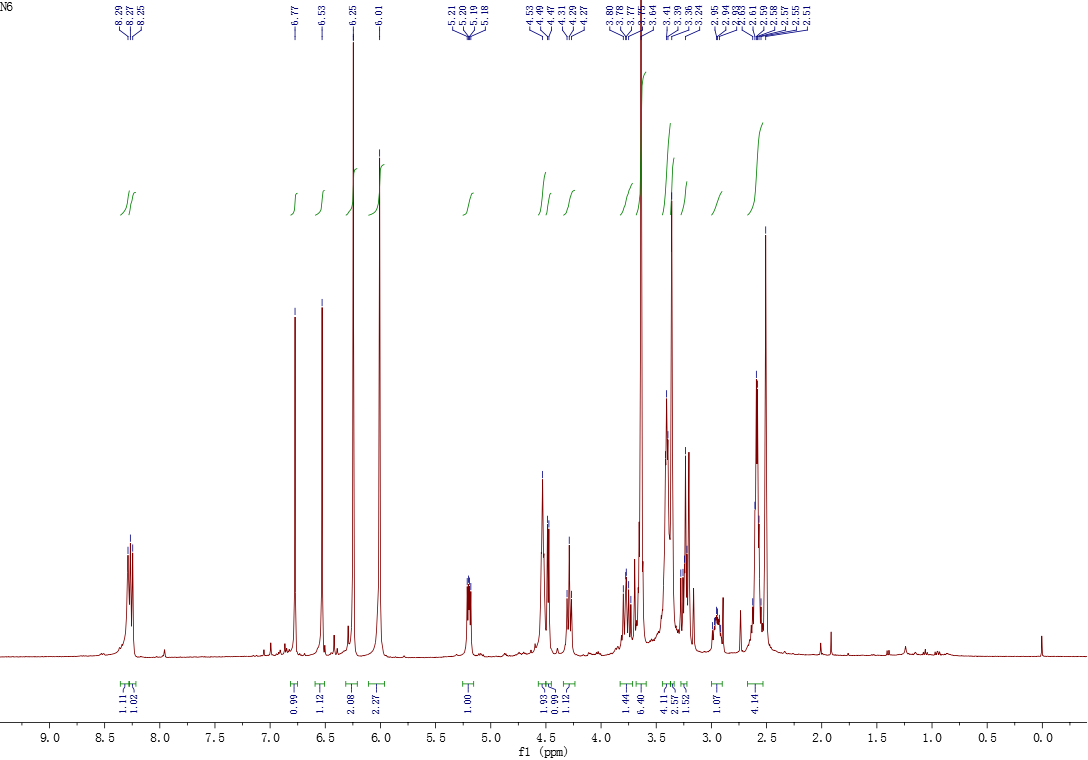
**

**^1^H NMR**

**
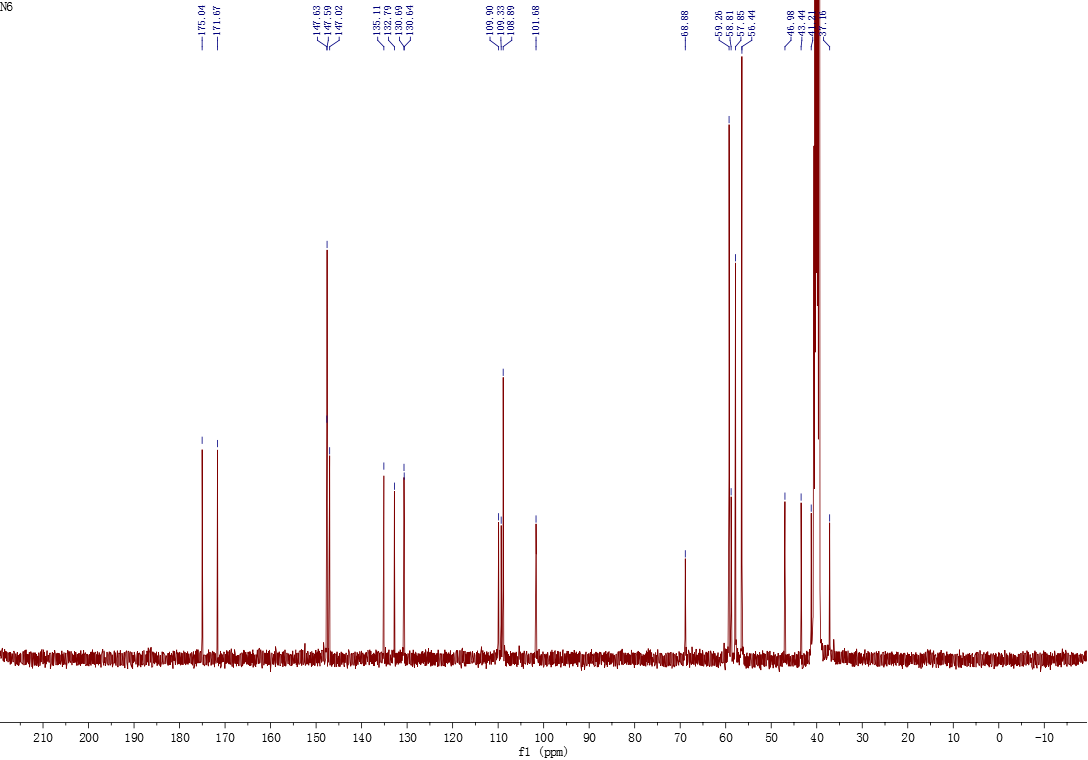
**

**^13^C NMR**

**12m：**

**
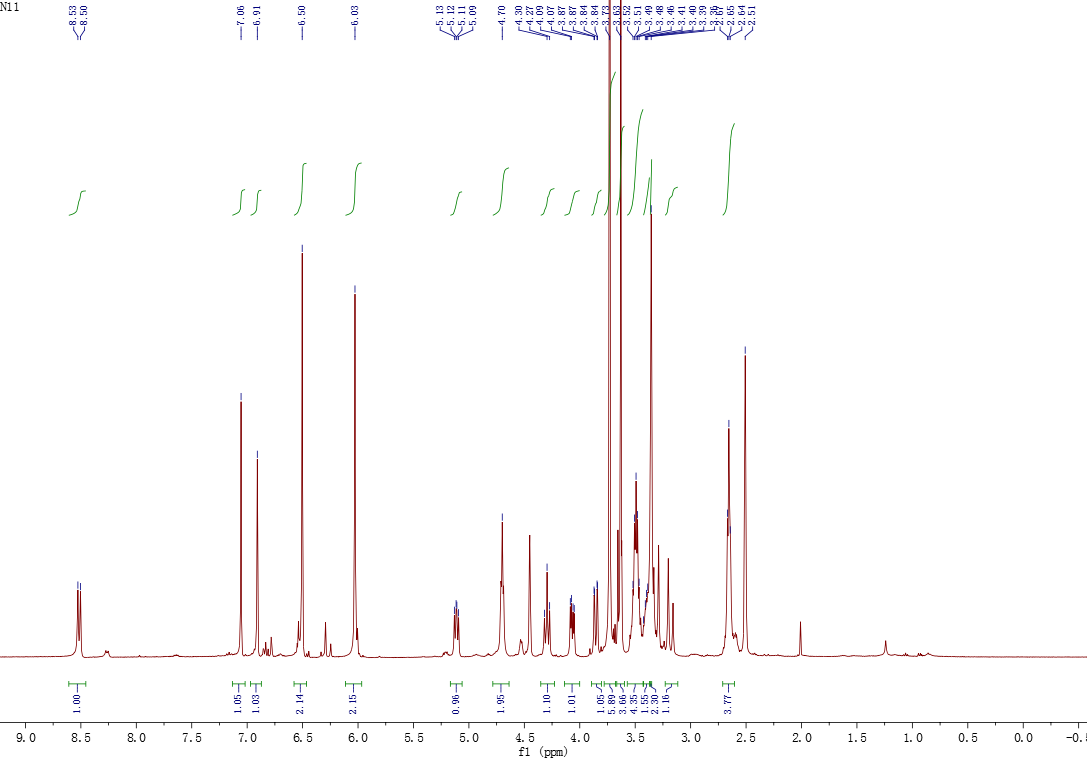
**

**^1^H NMR**

**
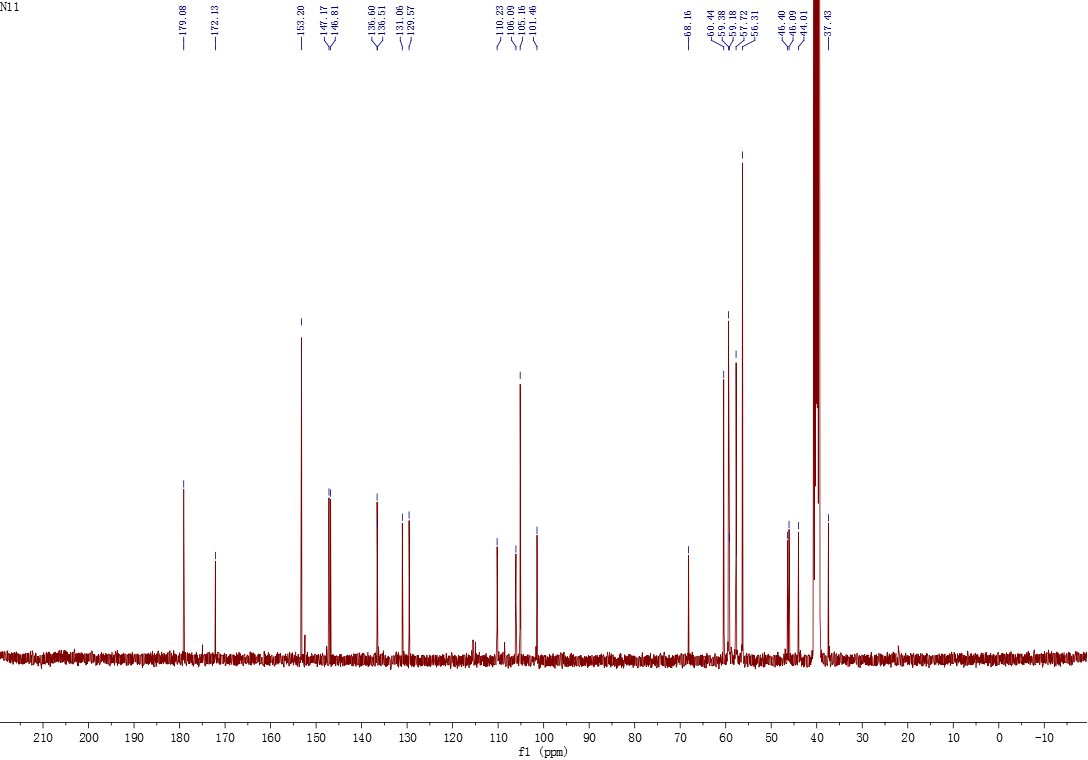
**

**^13^C NMR**

**12n：**

**
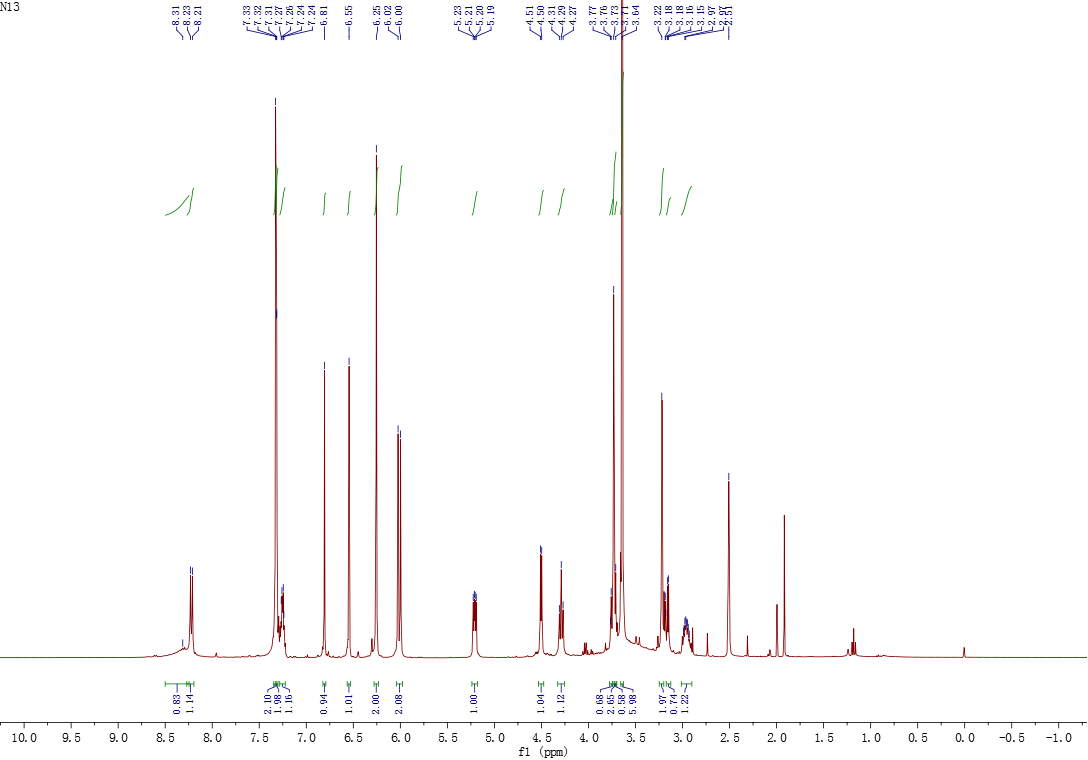
**

**^1^H NMR**

**
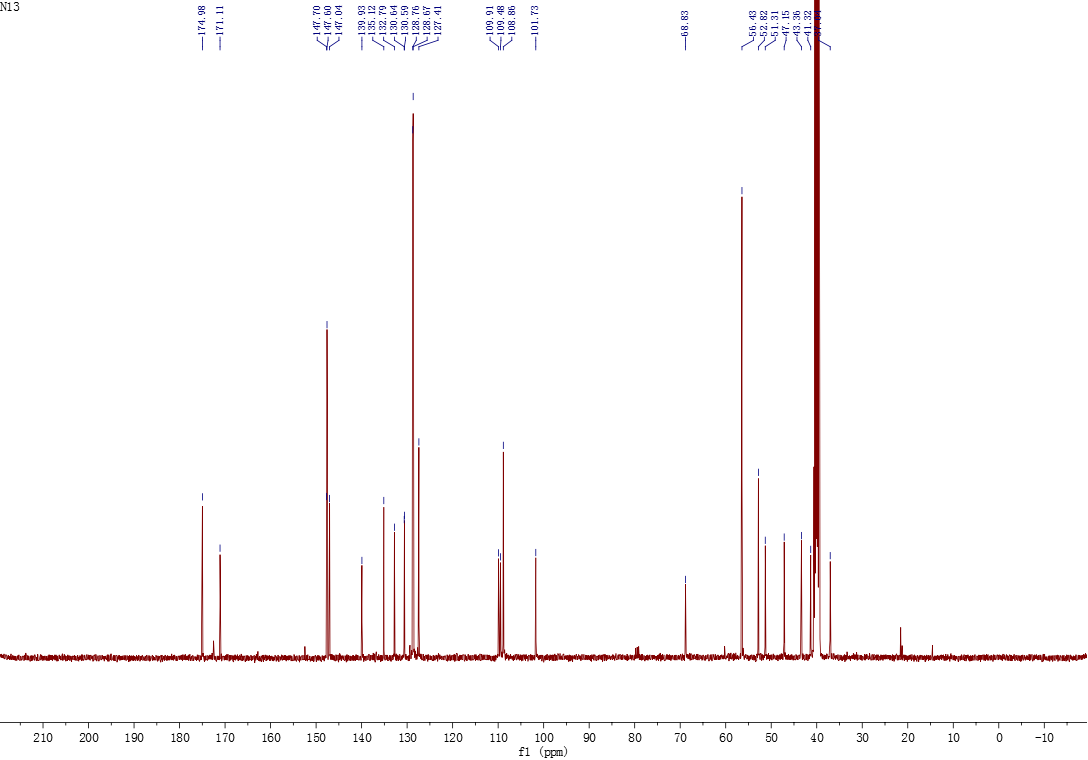
**

**^13^C NMR**

**12o：**

**
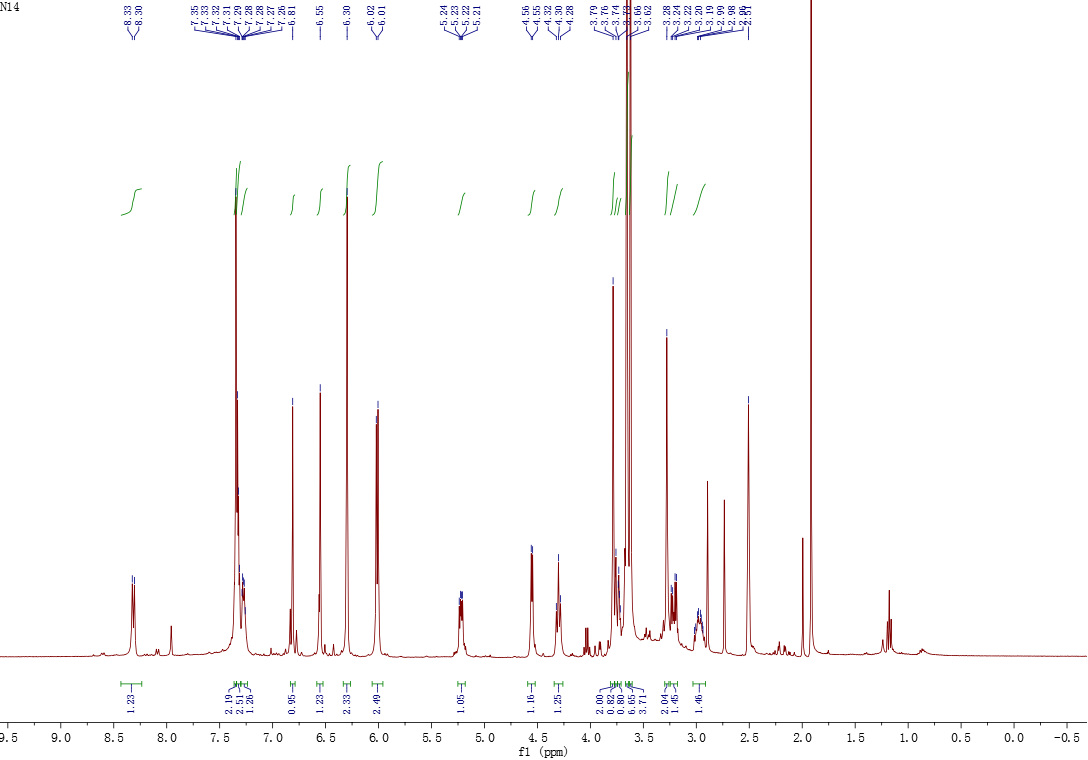
**

**^1^H NMR**

**
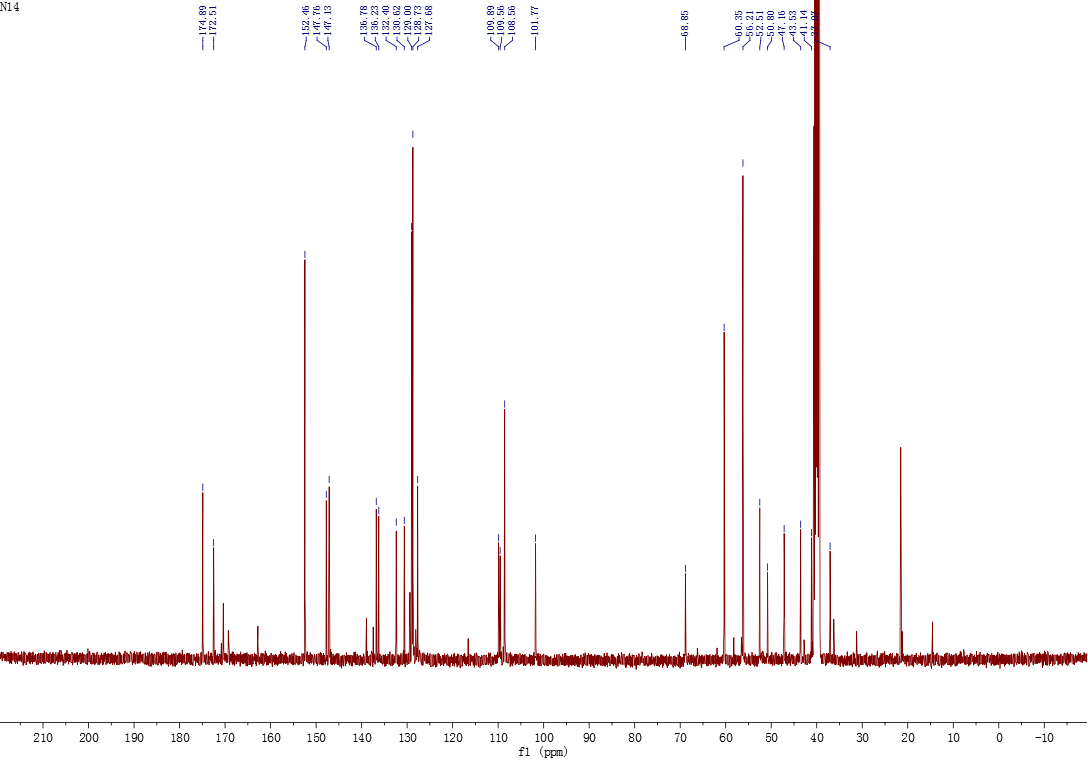
**

**^13^C NMR**

**12p：**

**
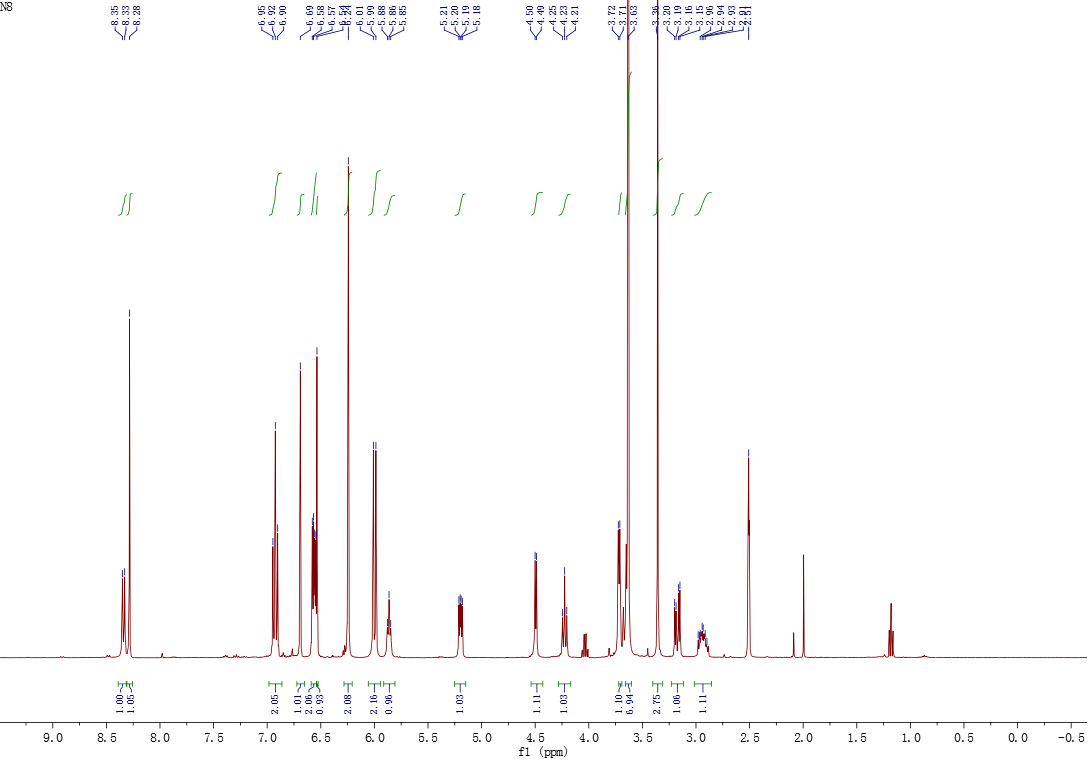
**

**^1^H NMR**

**
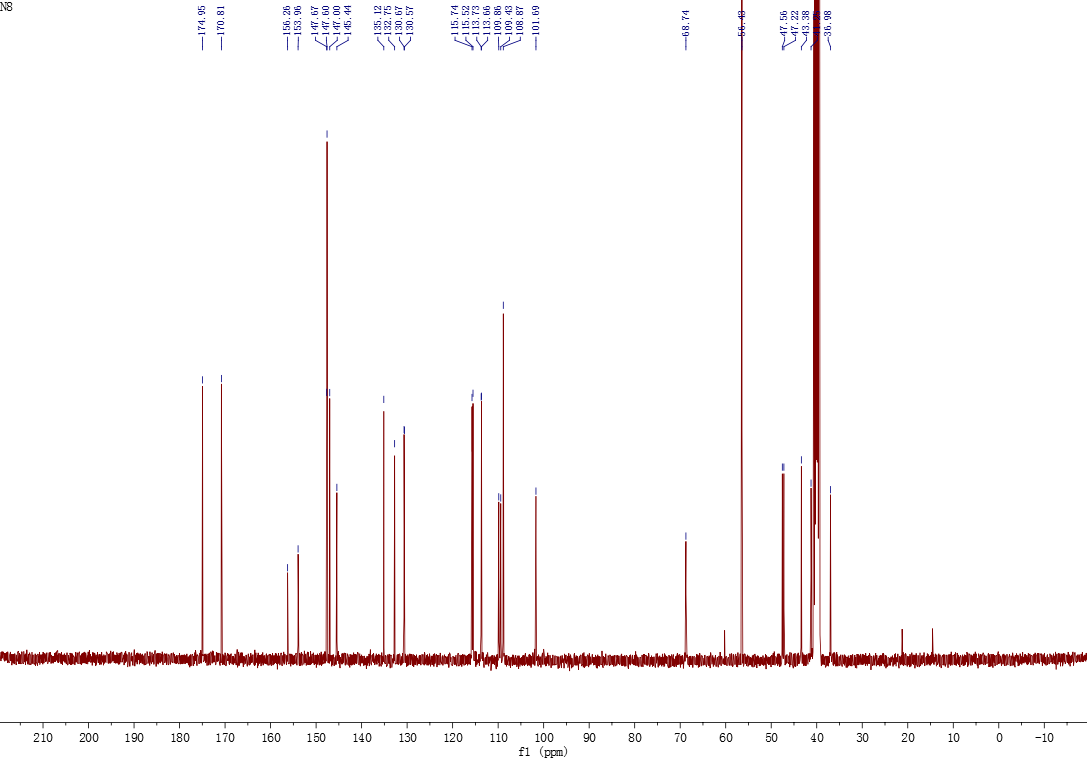
**

**^13^C NMR**

**12q：**

**
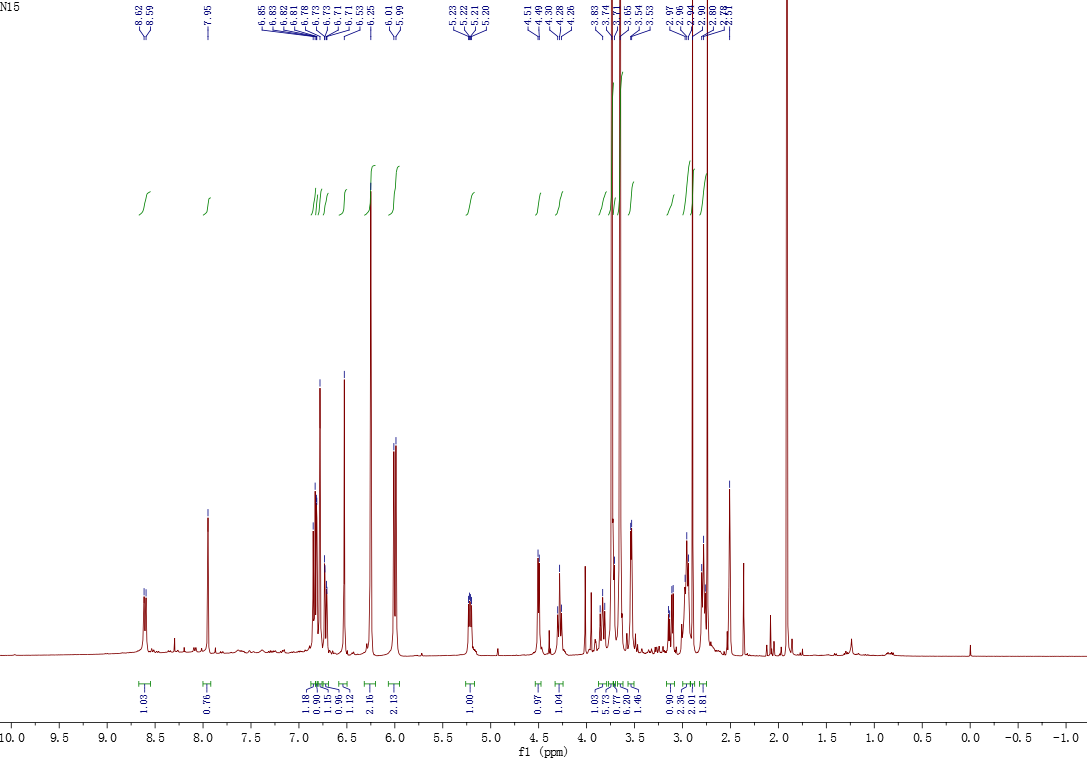
**

**^1^H NMR**

**
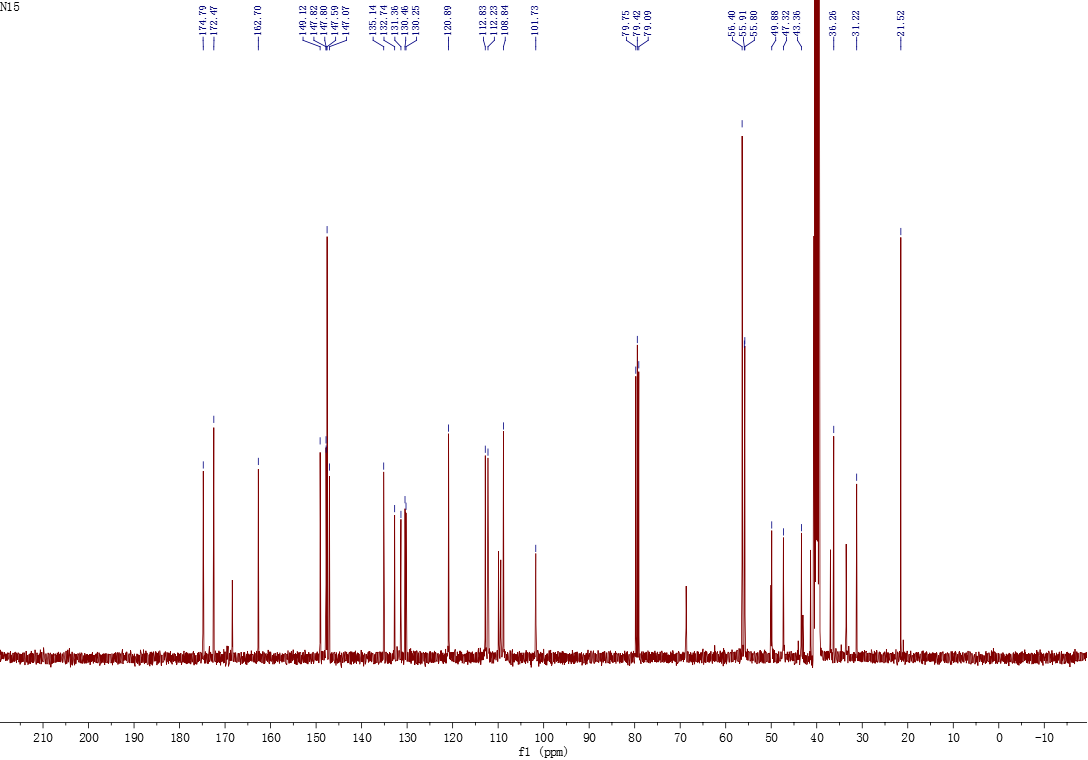
**

**^13^C NMR**

**12r:**

**
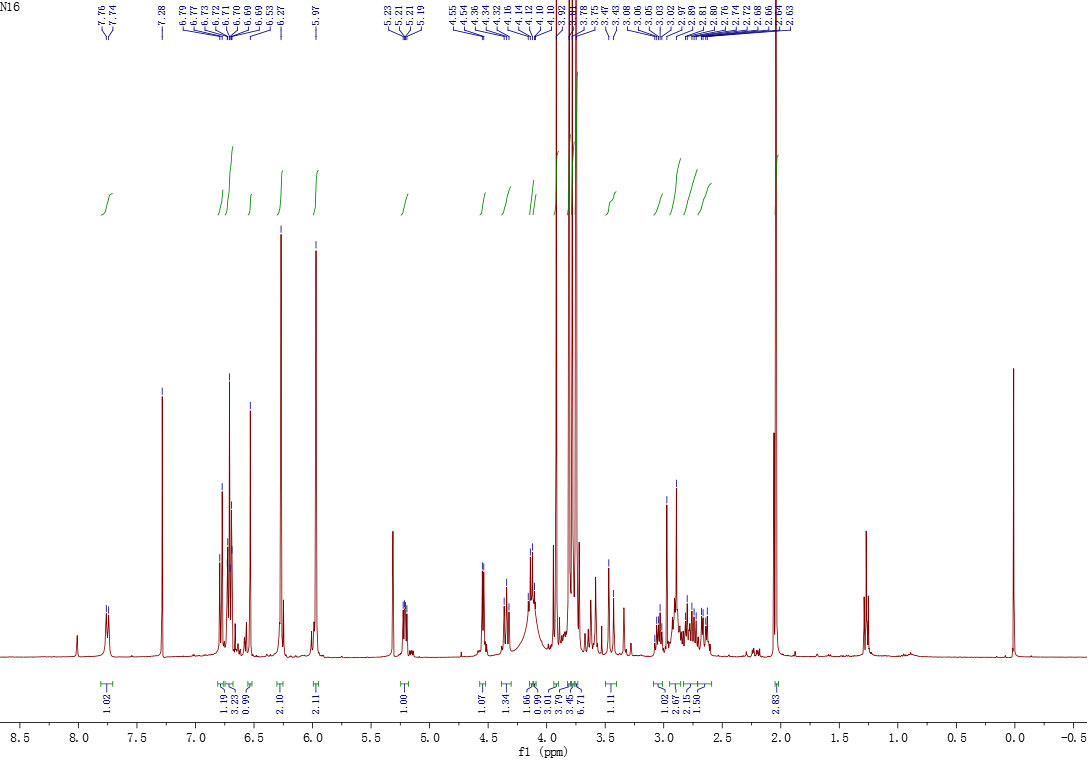
**

**^1^H NMR**

**
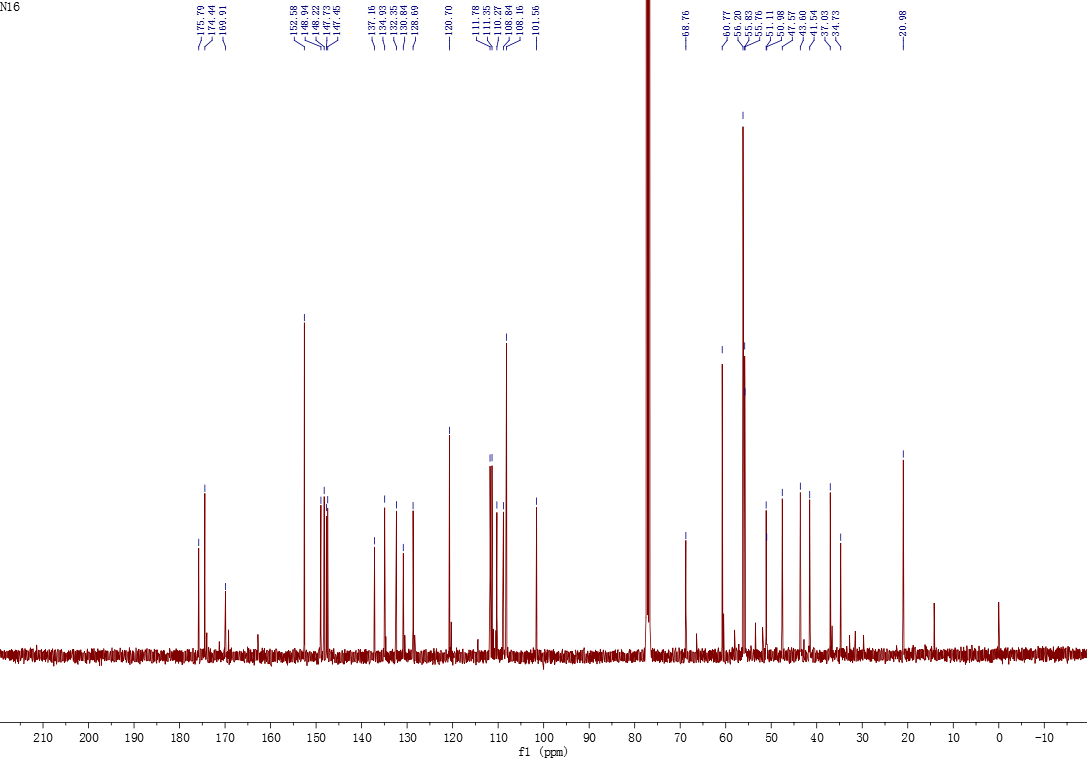
**

**^13^C NMR**

**12s：**

**
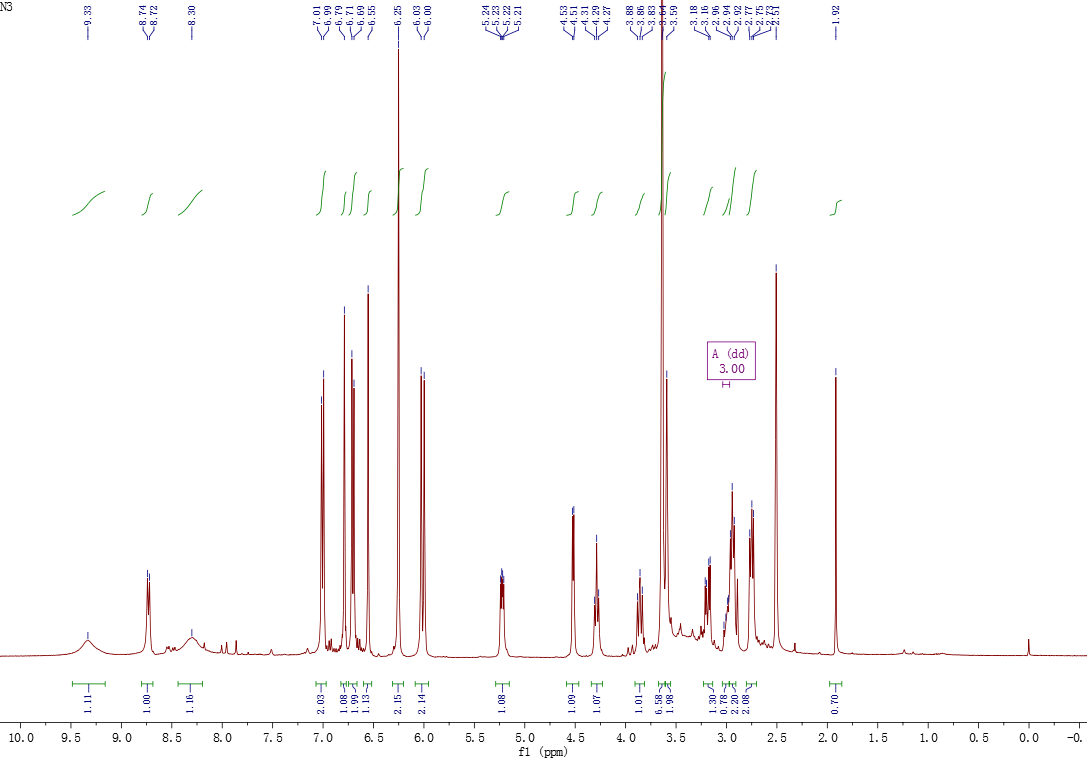
**

**^1^H NMR**

**
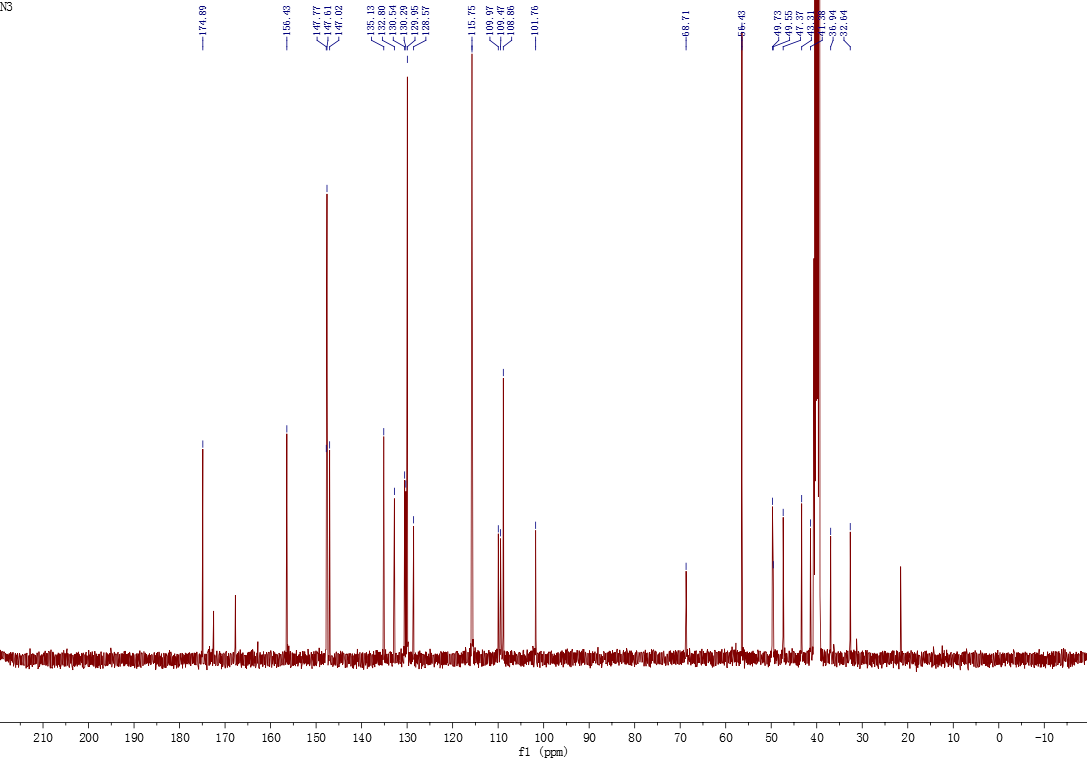
**

**^13^C NMR**

**12t:**

**
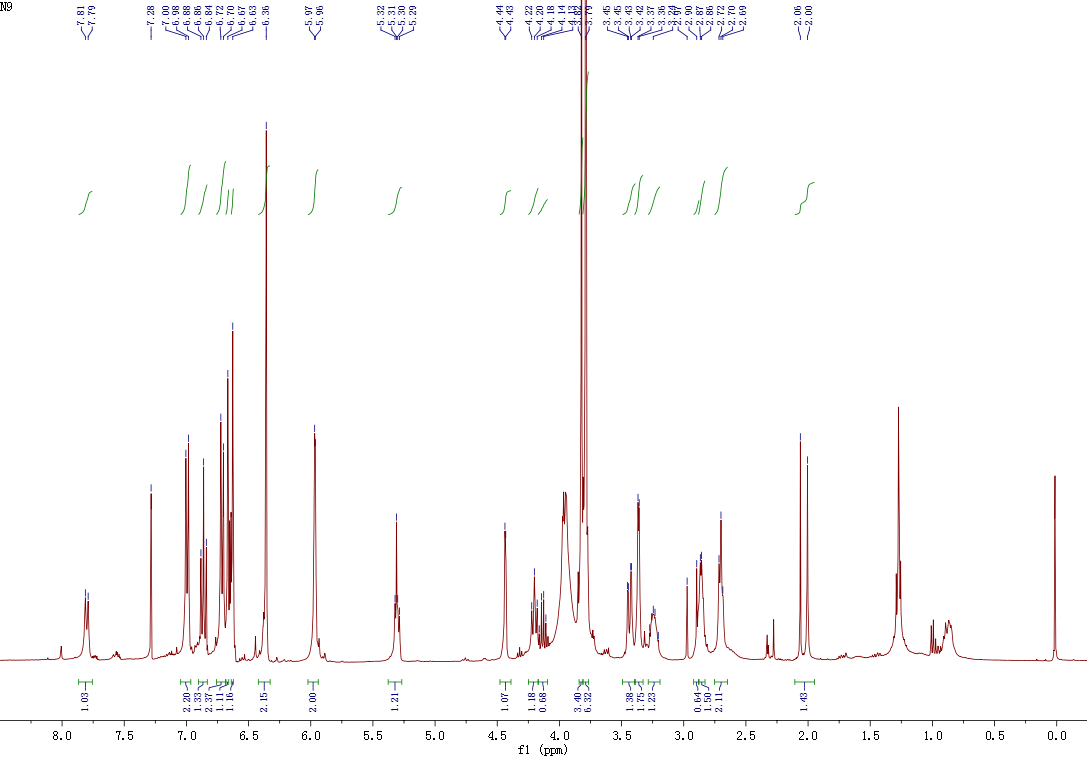
**

**^1^H NMR**


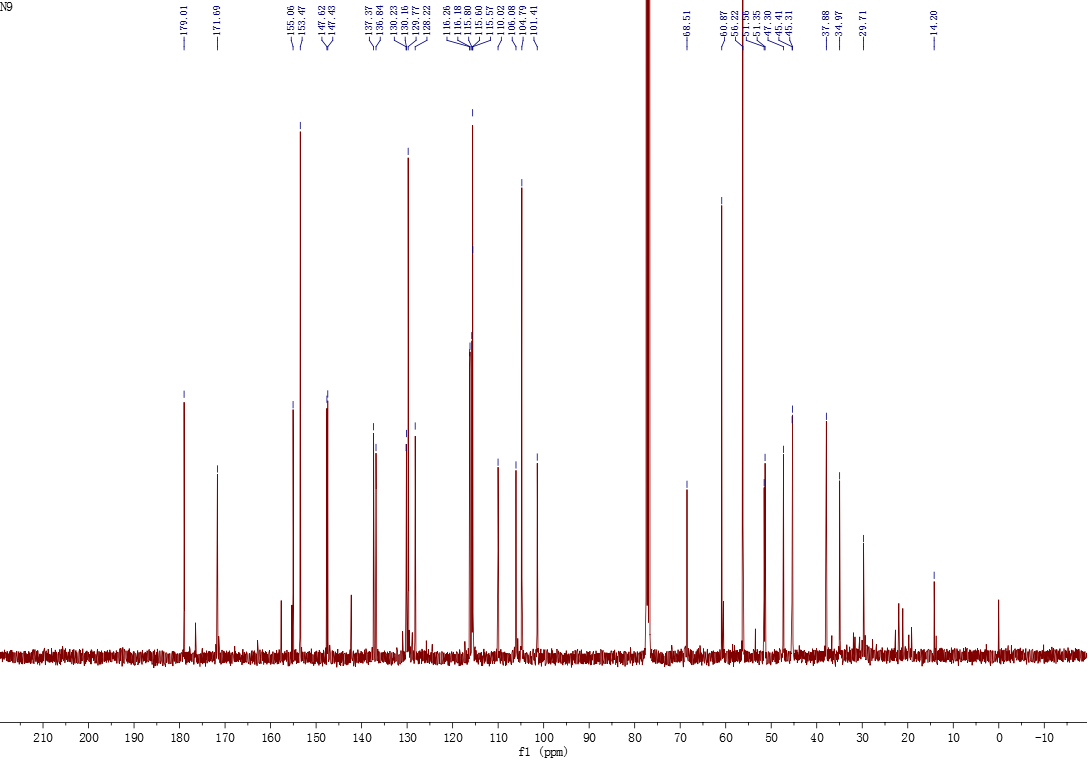


**^13^C NMR**
